# The senescent secretome drives PLVAP expression in cultured human hepatic endothelial cells to promote monocyte transmigration

**DOI:** 10.1016/j.isci.2023.107966

**Published:** 2023-09-19

**Authors:** Alex L. Wilkinson, Samuel Hulme, James I. Kennedy, Emily R. Mann, Paul Horn, Emma L. Shepherd, Kelvin Yin, Marco Y.W. Zaki, Gareth Hardisty, Wei-Yu Lu, Pia Rantakari, David H. Adams, Marko Salmi, Matthew Hoare, Daniel A. Patten, Shishir Shetty

**Affiliations:** 1 Centre for Liver and Gastrointestinal Research, Institute of Immunology and Immunotherapy, University of Birmingham, Birmingham B15 2TT, UK; 2 University of Cambridge, Cancer Research UK Cambridge Institute, Robinson Way, Cambridge CB2 0RE, UK; 3 Department of Biochemistry, Faculty of Pharmacy, Minia University, Minia, Egypt; 4 Centre for Inflammation Research, University of Edinburgh, Edinburgh EH8 9YL, UK; 5 Institute of Biomedicine, University of Turku, Turku, Finland; 6 MediCity Research Laboratory, University of Turku, Turku, Finland; 7 University of Cambridge, Department of Medicine, Addenbrooke’s Hospital, Cambridge CB2 0QQ, UK; 8 National Institute for Health Research, Birmingham Biomedical Research Centre at University Hospitals Birmingham NHS Foundation Trust, Birmingham, UK; 9 College of Health and Life Sciences, Aston University, Birmingham B4 7ET, UK

**Keywords:** Microenvironment, Molecular biology, Cell biology, Omics, Transcriptomics

## Abstract

Liver sinusoidal endothelial cells (LSEC) undergo significant phenotypic change in chronic liver disease (CLD), and yet the factors that drive this process and the impact on their function as a vascular barrier and gatekeeper for immune cell recruitment are poorly understood. Plasmalemma-vesicle-associated protein (PLVAP) has been characterized as a marker of LSEC in CLD; notably we found that PLVAP upregulation strongly correlated with markers of tissue senescence. Furthermore, exposure of human LSEC to the senescence-associated secretory phenotype (SASP) led to a significant upregulation of PLVAP. Flow-based assays demonstrated that SASP-driven leukocyte recruitment was characterized by paracellular transmigration of monocytes while the majority of lymphocytes migrated transcellularly. Knockdown studies confirmed that PLVAP selectively supported monocyte transmigration mediated through PLVAP’s impact on LSEC permeability by regulating phospho-VE-cadherin expression and endothelial gap formation. PLVAP may therefore represent an endothelial target that selectively shapes the senescence-mediated immune microenvironment in liver disease.

## Highlights

• PLVAP expression in human liver disease correlates with markers of tissue senescence
• Human liver endothelium upregulates PLVAP when exposed to the SASP *in vitro*
• PLVAP mediates endothelial barrier function by regulating phospho-VE cadherin levels
• PLVAP supports paracellular transmigration of monocytes in SASP-treated endothelium

## Graphical abstract

**Figure:**
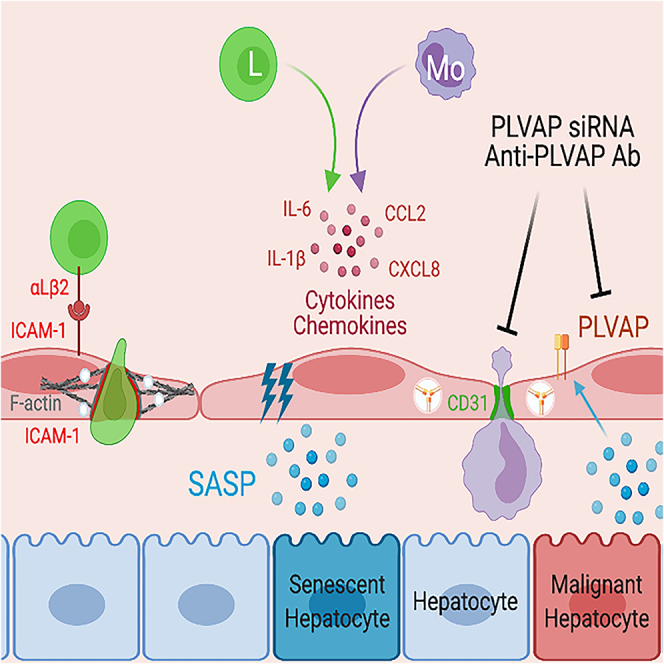

## Introduction

Chronic liver disease (CLD) is a global health burden accounting for approximately two million deaths/year worldwide.^1^^,^^2^ Ranking the second most common cause of premature death in the UK, CLD is also a major risk factor for developing hepatocellular carcinoma (HCC), which is predicted to affect >1 million individuals per year globally by 2025.^2^^,^^3^^,^^4^ CLDs are characterized by leukocyte infiltration, which drives chronic inflammation and fibrosis independently of etiology. The recruitment of immune cells takes place within the hepatic sinusoids that are lined by highly specialized fenestrated endothelia that act as the liver gatekeepers.^5^^,^^6^ The distinct phenotype of liver sinusoidal endothelial cells (LSEC), paired with the low shear environment within the hepatic sinusoids, fosters a unique environment in which leukocyte recruitment can occur. As such, it has become clear that the mechanisms that mediate this recruitment in the liver are distinct from more conventional vascular beds; understanding this process is critical to identify novel therapeutic targets that could allow selective manipulation of the hepatic immune microenvironment in CLD to promote wound healing and reduce cancer risk.

Plasmalemma-vesicle-associated protein (PLVAP) is the antigen recognized by PAL-E (pathologische anatomie Leiden-endothelium) and MECA-32 (mouse endothelial cell antigen-32) antibodies used to identify vascular endothelium in human and mouse tissues, respectively.^7^^,^^8^ It has long been considered an endothelial-specific protein, forming homodimeric diaphragms that span the openings of fenestrae and caveolae.^9^^,^^10^^,^^11^ In addition to its role in development, vascular permeability, and angiogenesis, as a component of these diaphragms, several studies have also implicated PLVAP in leukocyte trafficking.^12^^,^^13^^,^^14^ In particular, one study identified that PLVAP in lymphatic endothelium is important for lymphocyte entry into the lymph nodes,^13^ whereas another study indicated that PLVAP is integral for the egress of fetal liver monocytes and subsequent seeding as tissue-resident macrophages during development.^14^

Notably, recent single-cell RNA sequencing studies in humans have highlighted the re-emergence of PLVAP in diseased endothelium, such as within scar-associated endothelia of cirrhotic patients^15^ and tumor endothelia of HCC patients.^16^ Despite this, the regulation and functional role of PLVAP in liver disease is still poorly understood. Here, we confirmed that PLVAP is indeed upregulated in human liver cirrhosis, but for the first time, to our knowledge, demonstrate a direct correlation with cellular senescence within liver tissue.

In the context of both CLD and HCC, hepatocytes have previously been shown to reside in a state of cellular senescence,^17^ a process associated with production of a distinct secretome known as the senescence-associated secretory phenotype (SASP). The SASP has been characterized to comprise numerous cytokines, chemokines, growth factors, extracellular matrix proteins, and extracellular vesicles that are important in shaping the senescence tissue microenvironment.^18^ Importantly, the SASP is thought to drive recruitment of leukocytes to facilitate clearance of pre-malignant senescent cells, and indeed previous work suggests that monocytes and CD4^+^ T lymphocytes are critical in this process.^19^^,^^20^ We have previously demonstrated that SASP-stimulated liver endothelial cells support recruitment of peripheral blood lymphocytes under physiological shear conditions,^21^ but the molecular mechanisms of SASP-mediated leukocyte recruitment have yet to be completely elucidated. Here, we demonstrate that monocytes and lymphocytes transmigrate across SASP-stimulated LSEC using distinct pathways, identifying a specific role for PLVAP in monocyte recruitment. Furthermore, we utilize RNA sequencing, immunostaining, and transendothelial electrical resistance (TEER) assays to show that PLVAP supports paracellular migration by altering endothelial junctional integrity.

## Results

### PLVAP is upregulated within scar-associated endothelium in a range of human chronic liver diseases

**Figure 1:**
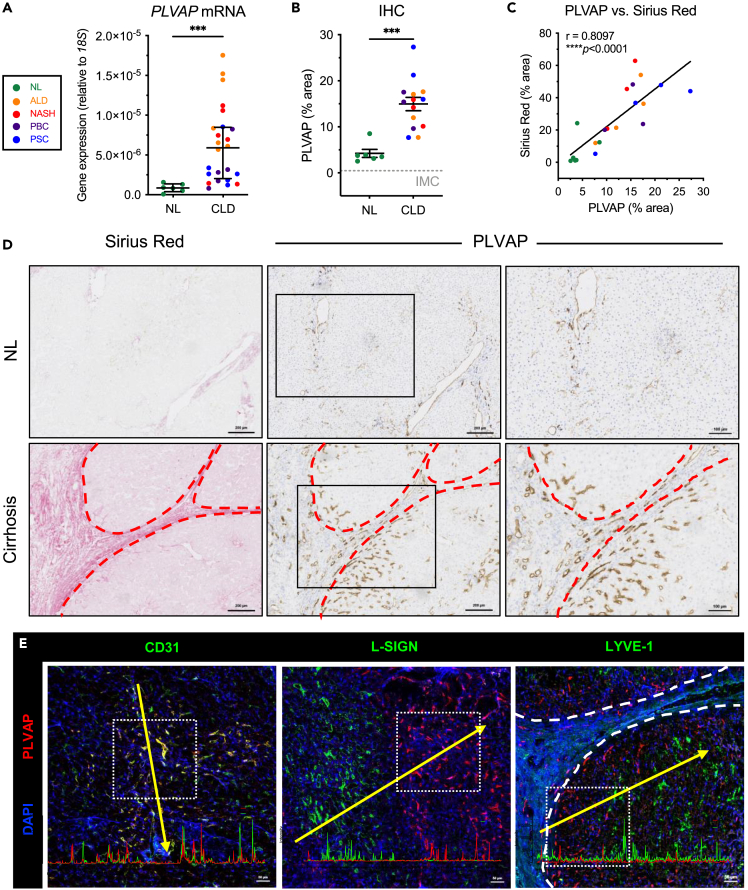
Plasmalemma-vesicle-associated protein (PLVAP) is upregulated in chronic liver disease (CLD) and displays a scar-associated expression pattern (A) *PLVAP* gene expression in normal liver (NL) (n = 6) versus cirrhotic liver (n = 24) was measured relative to *18S* by qRT-PCR. Data shown are median ± IQR (∗∗∗p < 0.001, Mann-Whitney test). (B) Quantification of PLVAP immunohistochemical (IHC) staining (% area) in NL (n = 6) and CLD (n = 14) tissue (mean ± SEM, ∗∗∗p < 0.001 Student’s unpaired t test). Isotype-matched control (IMC) level is indicated by the gray gridline. (C) Correlation between PLVAP and Sirius Red staining (% area) in matched patient samples (∗∗∗∗p < 0.0001, Pearson’s correlation test). (D) Representative IHC images of PLVAP and Sirius Red in matched serial sections from normal *(upper)* and cirrhotic *(lower)* human liver. Fibrotic septa are indicated by red dashed lines. Scale bars: 200 μm left and middle column, 100 μm right column. (E) Dual immunofluorescent staining of PLVAP (red) with CD31 (*left*), L-SIGN (*middle*), and LYVE-1 (*right*) (green) in cirrhotic liver. DAPI (blue) was used as a nuclear counterstain. Yellow lines depict site of the intensity profiles (*lower*). Scale bars: 50 μm.

Analysis of *PLVAP* mRNA in whole tissue lysates from normal and cirrhotic liver demonstrated a 6.9-fold upregulation of *PLVAP* gene expression across several CLDs, including alcoholic liver disease (ALD), non-alcoholic steatohepatitis (NASH), primary biliary cholangitis (PBC), and primary sclerosing cholangitis (PSC) (Figure 1A). Quantification of PLVAP staining confirmed its significant upregulation (3.5-fold), which occurred in CLD, irrespective of the liver etiology (Figure 1B). Furthermore, a positive correlation was observed between PLVAP and collagen deposition, as measured by Sirius red staining, in serial liver sections from the same patient (Figure 1C). Western blot analysis with an anti-PLVAP antibody detected a single band of ∼30 kDa that was largely absent from normal liver but significantly increased (7.9-fold) in CLD samples (Figure S1A). Immunohistochemical staining demonstrated that PLVAP^+^ vessels localized within fibrotic tracts (visualized using Sirius red) and also in the peri-fibrotic sinusoids (Figures 1D and S1B).

We next performed further spatial and phenotypic characterization of the PLVAP^+^ cell population by dual immunofluorescent staining with endothelial and sinusoidal markers. In cirrhotic liver, PLVAP co-localized with classical vascular marker, CD31, while displaying a mutually exclusive expression pattern with proteins enriched in LSEC, including liver/lymph-node-specific intercellular adhesion molecule-3-grabbing integrin (L-SIGN) and lymphatic vessel endothelial hyaluronan receptor 1 (LYVE-1) (Figures 1E and S2A). These data were in keeping with previous single-cell sequencing studies, highlighting an enrichment of *PLVAP* gene expression in endothelial cells that display a scar-associated genetic signature (Figure S2B).^15^ Consistent with our findings, publicly available single-cell sequencing data showed endothelial expression of *CLEC4M* (L-SIGN) and *LYVE1* in cells annotated as LSEC, in which *PLVAP* was largely absent (Figures S2C and S2D). Moreover, *PLVAP* was often co-expressed with *PECAM1* (CD31) in cirrhotic human liver endothelium (Figures S2C and S2D). Collectively, these data confirm that PLVAP is upregulated in human CLD and suggest that it defines a distinct scar-associated endothelial cell subset that may contribute to disease pathogenesis.

### PLVAP correlates with markers of cellular senescence and immune infiltrate in CLD

**Figure 2:**
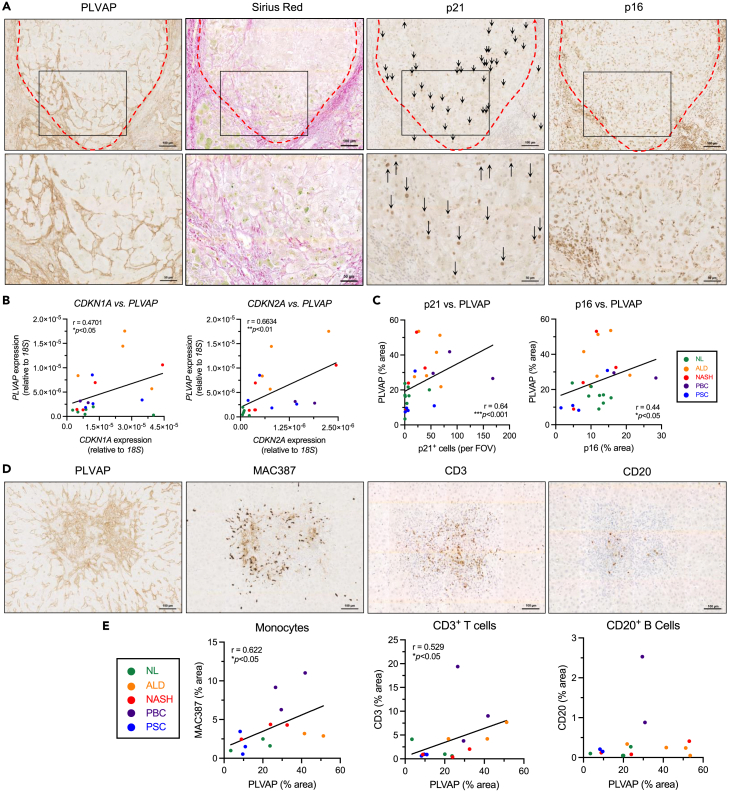
Plasmalemma-vesicle-associated protein (PLVAP) correlates with senescence and immune infiltrate in chronic liver disease (CLD) (A) Representative low (*upper*) and high (inset, *lower*) power images of immunohistochemical staining of PLVAP, Sirius Red, and senescence markers, p21 and p16, in serial sections from matched CLD patient samples. Scale bars: 100 μm top row, 50 μm bottom row. (B) Correlation analysis of *PLVAP* versus p21 (*CDKN1A*) (*left*) and *PLVAP* versus p16 (*CDKN2A*) *(right*) mRNA levels in normal liver (NL) (n = 5) and CLD (n = 15). Gene expression was measured relative to *18S* by qPCR (∗p < 0.05, ∗∗p < 0.01, Spearman’s correlation test). (C) Correlation analysis of PLVAP versus p21 (*left*) (n = 27) and PLVAP versus p16 (*right*) (n = 24) immunohistochemical staining in normal liver (NL) and CLD (∗p < 0.05, ∗∗∗p < 0.001, Spearman’s correlation test). (D) Representative immunohistochemical staining of PLVAP, MAC387 (infiltrating monocytes), CD3 (T cells), and CD20 (B cells) (*from left to right*) in serial sections from cirrhotic liver patient samples. Visual fields are the same for each marker. Scale bars: 100 μm. (E) Correlation analysis of PLVAP staining area (%) with MAC387, CD3, and CD20 (*from left to right*) in normal liver (NL) (n = 3) and CLD (n = 11–12) (∗p < 0.05, Spearman’s correlation test).

Previous studies have shown that hepatocytes peripheral to fibrotic tracts in CLD tissues largely exist in a senescent state.^22^^,^^23^^,^^24^^,^^25^ Given that PLVAP is enriched within these same regions, we sought to investigate a potential link between PLVAP expression and hepatic senescence. Immunohistochemistry was performed on serial liver sections from the same patient to visualize PLVAP in conjunction with fibrotic regions (Sirius red), along with senescence markers, p21 and p16 (Figure 2A). From our immunohistochemical staining we could clearly identify p21^+^ hepatocytes and found a significant increase in p21^+^ hepatocytes in CLD compared with normal liver, quantified by positive cell per field of view (Figures S3A and S3B). We also noted that the enrichment of PLVAP within peri-fibrotic areas spatially coincided with p21^+^ hepatocytes (Figure 2A). In contrast, we found p16 staining was highly variable in cirrhotic specimens (Figures S3A and S3B). p16^+^ hepatocytes staining was relatively more homogeneous in their distribution throughout regenerative nodules; localization of other p16^+^ cells (possibly immune cells) was observed within fibrotic septa in close association with PLVAP-enriched areas (Figure 2A) with total expression quantified as % area. Further analysis demonstrated that PLVAP was directly proportional to expression of p21 and p16 in matched human liver samples, at both the gene and the protein level, as determined by qRT-PCR (Figure 2B) and quantification of immunohistochemical staining (Figure 2C), respectively. These data highlight a previously unreported link between PLVAP expression and a senescent hepatic microenvironment in CLD.

Senescent cells release a secretome known as the senescence-associated secretory phenotype (SASP), which can regulate the immune microenvironment by driving leukocyte recruitment.^19^^,^^21^^,^^26^ Given the distribution of PLVAP^+^ endothelial cells within peri-fibrotic areas, which are frequent sites of leukocyte recruitment during chronic inflammation, we hypothesized that PLVAP could be a critical link between senescence and the immune microenvironment within the liver. We undertook immunohistochemistry of serial liver sections to visualize PLVAP with respect to immune cell infiltration; specifically, infiltrating monocytes (MAC387), T lymphocytes (CD3), and B lymphocytes (CD20) were explored. MAC387 is known to recognize both monocytes and neutrophils,^27^^,^^28^^,^^29^ and so neutrophil elastase (NE) staining was performed in matched cases. The levels of infiltrating neutrophils were minimal in all cases tested, suggesting their contribution to MAC387 positivity was negligible (Figure S3C). In normal liver, lymphocyte infiltration was minimal, whereas MAC387^+^ cells were found to be homogenously distributed throughout the sinusoids (Figure S3D). In contrast, in cirrhotic liver, PLVAP-rich areas marked the sites of extensive MAC387^+^, CD3^+^, and CD20^+^ cell infiltration (Figure 2D). Moreover, PLVAP (% area) positively correlated with MAC387 and CD3 (but not CD20) immunostaining in matched patient samples (Figure 2E), suggesting PLVAP may be associated with recruitment of monocytes and T lymphocytes in CLD.

### PLVAP expression is maintained in primary human LSEC and is upregulated by the senescent secretome *in vitro*

**Figure 3:**
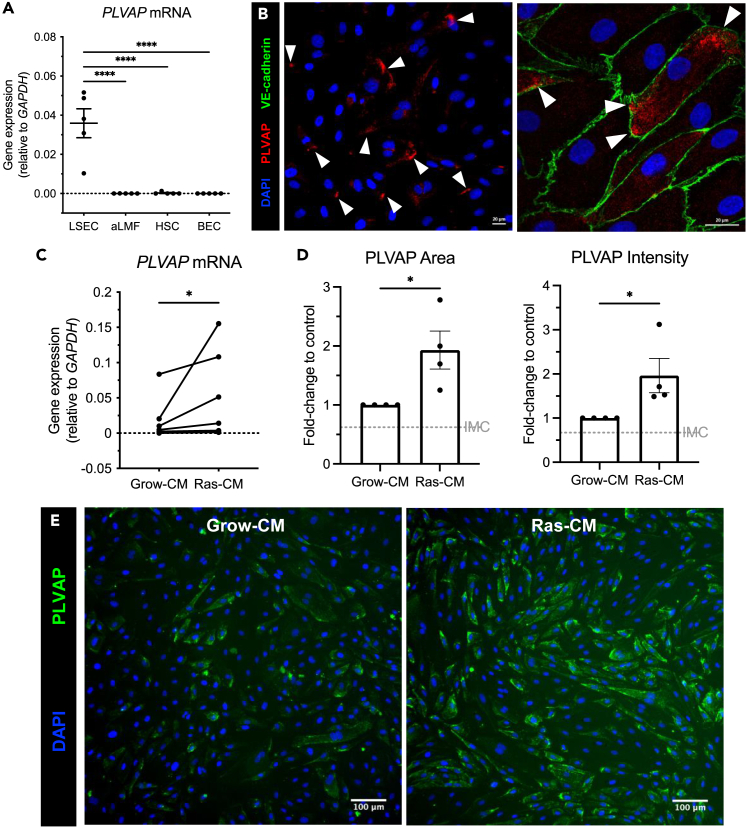
Plasmalemma-vesicle-associated protein (PLVAP) is maintained *in vitro* in primary liver sinusoidal endothelial cells (LSEC) and is upregulated by the senescent secretome (A) *PLVAP* gene expression in passaged LSEC, activated liver myofibroblasts (aLMF), hepatic stellate cells (HSC), and biliary epithelial cells (BEC) relative to *GAPDH* (n = 5). Data shown are mean ± SEM (∗∗∗∗p < 0.0001, one-way ANOVA followed by Holm-Šídák’s multiple comparisons test). (B) Confocal images of PLVAP (red) immunofluorescence (white arrowheads) in patient-derived LSEC (25x objective) (*left*) and with junctional marker, VE-cadherin (green) (*right*) (63× objective). DAPI (blue) was used as a nuclear counterstain. Scale bars: 20 μm. (C) *PLVAP* gene expression relative to *GAPDH* in LSEC following 24 h treatment with the senescent secretome (Ras-CM) or the growing control (Grow-CM) (∗p < 0.05, Wilcoxon test) (n = 7). (D) PLVAP immunofluorescence area (*left*) and intensity (*right*) following Grow-CM or Ras-CM treatment. Staining was quantified via high-content imaging where nine visual fields per well were analyzed, with each condition performed in at least duplicate. Data shown are mean ± SEM from four independent cell isolates (∗p < 0.05, Student’s unpaired t test (area) or Mann-Whitney test (intensity). Isotype-matched control (IMC) levels are indicated by the gray gridline. (E) Representative immunofluorescent images of PLVAP (green) in LSEC following 24 h Grow-CM or Ras-CM treatment. DAPI (blue) was used as a nuclear counterstain. Scale bars:100 μm.

To investigate whether PLVAP expression is maintained *in vitro*, primary LSEC were isolated and *PLVAP* gene expression was compared with other cultured non-parenchymal hepatic cell types. *PLVAP* mRNA levels were significantly higher in LSEC when compared with activated liver myofibroblasts (aLMF) (>2000-fold), hepatic stellate cells (HSC) (7400-fold), and biliary epithelial cells (BEC) (4700-fold) (Figure 3A). The expression of PLVAP was also maintained *in vitro* at the protein level in passaged LSEC, as determined by immunofluorescence (Figure 3B). Notably, PLVAP was expressed only in a subset of LSEC, recapitulating observations *in situ* in human cirrhotic liver. In these positive LSEC, PLVAP often localized toward the cell periphery, although it was excluded from the VE-cadherin^+^ intercellular junctions (Figure 3B).

As PLVAP expression was maintained in LSEC *in vitro*, this offered the opportunity to study the regulation of PLVAP expression in these cells. Given the distinct spatial localization of PLVAP relative to fibrotic septa, there are likely factors released and concentrated within these regions that are important for its paracrine regulation. As such, we developed a high-content imaging assay to probe the modulation of PLVAP in primary human LSEC in response to various stimuli (Figure S4A). To confirm the sensitivity and validity of this assay we initially treated LSEC with tumor necrosis factor alpha (TNF-α), which is known to upregulate intercellular adhesion molecule 1 (ICAM1) in liver endothelial cells in a dose-dependent manner^30^ (Figures S4B and S4C). Once optimized, the assay was repeated with vascular endothelial growth factor (VEGF), which has been shown to regulate PLVAP expression in multiple endothelial cell types.^31^^,^^32^^,^^33^^,^^34^^,^^35^ Our assay confirmed that PLVAP expression was upregulated in human LSEC by vascular endothelial growth factor (VEGF) treatment for 24 h, demonstrated by an increase in immunofluorescence area (1.8-fold ± 0.14) and intensity (1.9-fold ± 0.17) compared with the untreated control (Figures S4D and S4E).

Building on these regulation studies, we next explored factors that more accurately reflect the liver microenvironment *in vivo*. VEGF plays an important role in angiogenesis and is a master regulator of endothelial cell biology, yet is also known to be a key mediator in CLD and HCC.^36^^,^^37^ Hepatocytes are known to be a major source of VEGF within the liver,^38^^,^^39^^,^^40^ and we confirmed that a hepatocyte-endothelial axis could be important for PLVAP regulation in CLD, showing that supernatants from hepatocyte cell line, HepG2, also upregulated PLVAP in LSEC (Figures S4F and S4G). Given the correlation between PLVAP and senescence in CLD specimens, paired with the regulatory effects of VEGF and hepatoma cell supernatants, we aimed to model a senescent microenvironment *in vitro*. We used a well-established and validated model of oncogene-induced senescence, in which SASP was obtained from IMR90 human diploid fibroblasts overexpressing tamoxifen-inducible oncogenic *HRAS*^*G12V*^ (Ras-CM), and compared this with the effects of the growing, non-senescent cell control (“Grow-CM”). This model has provided key insights into cellular senescence within the liver.^21^ Ras-CM stimulation of LSEC for 24 h induced both PLVAP mRNA (3.0-fold) (Figure 3C) and protein expression (Figures 3D and 3E), significantly increasing immunofluorescence area (2.1-fold ± 0.19) and intensity (2.0-fold ± 0.34) in cultured LSEC. These data indicate that soluble factors released within a diseased tissue microenvironment may underpin the distinct expression of PLVAP.

### The senescent secretome drives recruitment of lymphocytes and monocytes by molecularly distinct mechanisms

**Figure 4:**
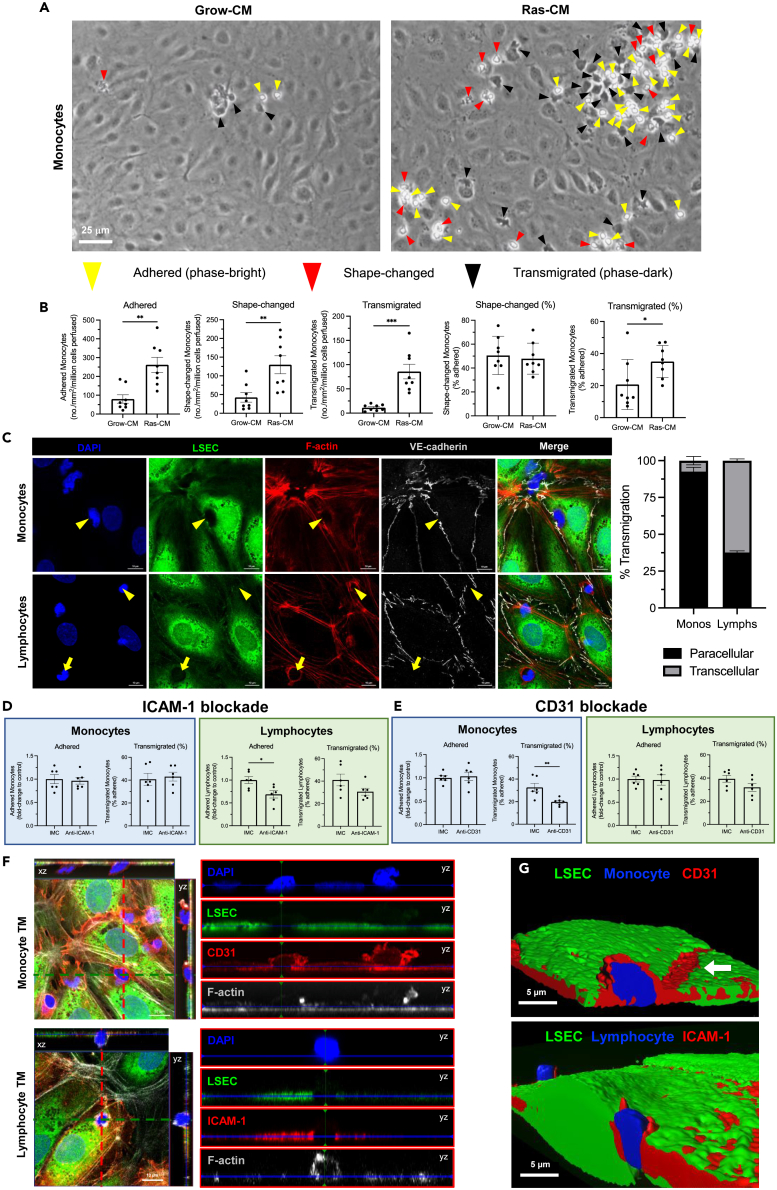
The senescent secretome drives recruitment of lymphocytes and monocytes across primary human liver sinusoidal endothelial cells (LSEC) by distinct molecular mechanisms (A) Flow adhesion assays were performed with peripheral blood monocytes and primary LSEC following Grow-CM or Ras-CM stimulation for 24 h. Representative phase-contrast images are shown indicating adhered (yellow arrowheads), shape-changed (red arrowheads), and transmigrated (black arrowheads) monocytes. Scale bar: 25 μm. (B) Quantification of adhered, shape-changed, and transmigrated monocytes following flow assays with Grow-CM- or Ras-CM-treated LSEC. Data shown are mean ± SEM from six independent experiments where 10 visual fields were analyzed per condition (∗p < 0.05, ∗∗p < 0.01, ∗∗∗p < 0.001, Mann-Whitney test (adhered and % transmigrated) or Student’s unpaired t test). (C) Confocal images of LSEC pre-labeled with CellTracker Green (green) and SiR-actin (red) following flow assays with monocytes (*upper*) or lymphocytes (*lower*). Paracellular (yellow arrowheads) and transcellular (yellow arrows) transmigration (TM) was determined based on integrity of VE-cadherin^+^ intercellular junctions (gray). Quantification of transmigratory route as a percentage of total TM events is shown (159 lymphocyte events and 327 monocyte events). Data are mean ± SEM from three independent cell isolates. Scale bars: 10 μm. (D) Quantification of adhered and transmigrated (% adhered) monocytes (*left*) and lymphocytes (*right*) following antibody-mediated blockade of intercellular adhesion molecule 1 (ICAM-1) (∗p < 0.05, Mann-Whitney test). (E) Quantification of adhered and transmigrated (% adhered) monocytes (*left*) and lymphocytes (*right*) following antibody-mediated blockade of CD31 (∗∗p < 0.01, Student’s unpaired t test). (F) Orthogonal confocal images of monocyte (*upper*) and lymphocyte (*lower*) TM. LSEC were pre-labeled with CTG (green) and SiR-actin (gray). CD31 (*upper*) and ICAM-1 (*lower*) were stained post-fixation (red). (G) 3D rendered images of z-stacks showing monocyte (*upper*) and lymphocyte (*lower*) TM in association with CD31 and ICAM-1, respectively (red). Scale bars: 10 μm left column, 5 μm right column.

SASP release is known to facilitate senescent cell surveillance by driving immune cell recruitment,^19^^,^^21^ and recently we have shown that LSEC play a critical role in this process.^41^ Here, we confirm that primary human LSEC undergo activation in response to 24-h Ras-CM treatment, including morphological changes such as elongation, cytoskeletal rearrangement and actin stress fiber formation (Figures S5A and S5B), and production of proinflammatory cytokines, chemokines, and adhesion molecules (Figures S5C–S5E). We have previously shown that SASP-stimulated liver endothelial cells support lymphocyte recruitment under physiological shear stress.^21^ To investigate whether SASP treatment can also drive monocyte recruitment *in vitro*, flow adhesion assays were performed with Grow-CM- or Ras-CM-stimulated LSEC and purified healthy peripheral blood monocytes. These flow-based adhesion assays imaged with phase-contrast microscopy recapitulate leukocyte recruitment within the hepatic sinusoids and permit analysis of each step of the adhesion cascade from leukocyte capture through to transendothelial migration.^42^^,^^43^^,^^44^ Monocyte adhesion (3.3-fold), shape change (activation) (3.1-fold), and transmigration (7.7-fold) were significantly higher following LSEC SASP exposure compared with control cells (Figures 4A and 4B). Therefore, SASP-stimulated liver endothelial cells are capable of recruiting both lymphocytes and monocytes under physiologically low shear conditions.

Next, we studied the mechanisms of innate versus adaptive immune cell recruitment in response to the SASP, with a focus on myeloid and lymphocyte populations because their infiltration into the liver is a hallmark of several CLDs. High-resolution imaging of fluorescently labeled LSEC by confocal microscopy revealed that SASP-primed monocyte and lymphocyte transmigration occurred via distinct endothelial routes. Specifically, >92% of monocytes transmigrated via the paracellular route in response to the SASP, which was determined by displacement of the VE-cadherin^+^ endothelial junctions (Figure 4C). In contrast, lymphocytes predominantly utilized the transcellular path (∼60%) during transmigration, in association with F-actin-rich transmigratory pores (Figure 4C).

In keeping with the differences in transmigratory route, SASP-mediated monocyte and lymphocyte recruitment were also found to be molecularly distinct. Antibody-mediated ICAM1 blockade had no observed effect on monocyte recruitment but reduced both lymphocyte adhesion (0.7-fold ± 0.08) and % transmigration (41.0%–30.6%) (Figure 4D). Furthermore, monocyte % transmigration was significantly impaired (32.6%–19.8%) following antibody-mediated CD31 inhibition, whereas monocyte and lymphocyte adhesion were unaffected by CD31 blockade (Figure 4E). Lymphocyte transmigration was slightly reduced in CD31-inhibited LSEC, 39.4%–32%, although this was not statistically significant (Figure 4E). Consistent with these findings, transmigrating lymphocytes and monocytes were observed to associate with ICAM1 and CD31, respectively, during SASP-mediated recruitment (Figures 4F and 4G). Thus, the senescent secretome drives endothelial recruitment of monocytes and lymphocytes by distinct molecular mechanisms.

### PLVAP contributes to SASP-mediated monocyte, but not lymphocyte, transmigration across primary human LSEC

**Figure 5:**
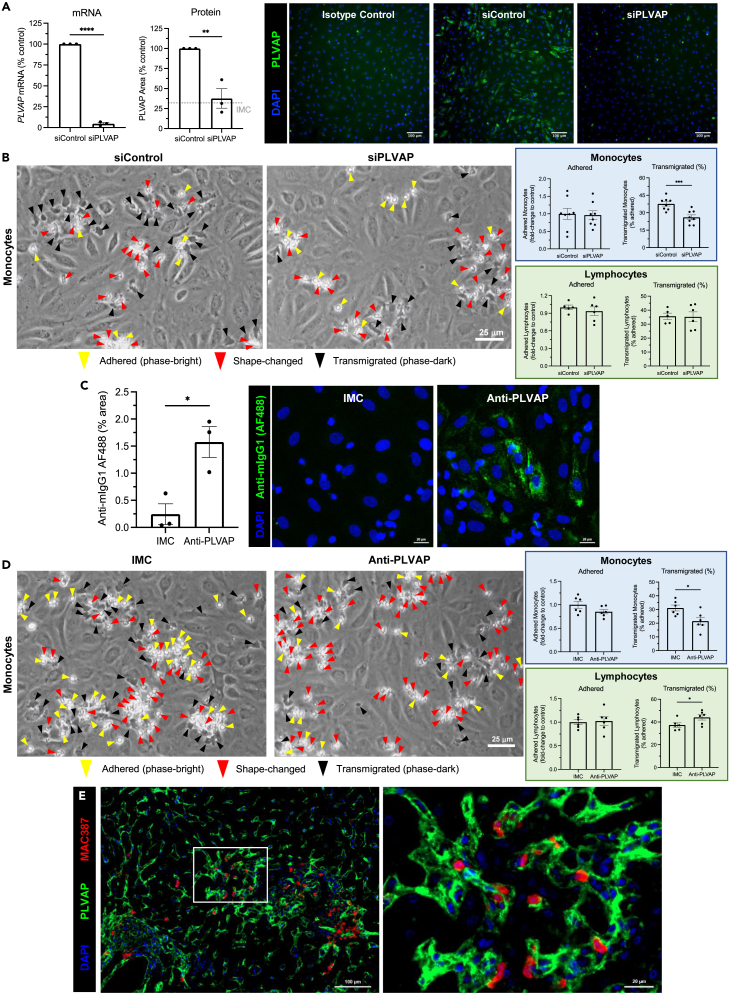
Plasmalemma-vesicle-associated protein (PLVAP) mediates monocyte, but not lymphocyte, transmigration across patient-derived liver sinusoidal endothelial cells (LSEC) in response to the senescent secretome (A) Genetic knockdown of PLVAP was performed via siRNA transfection of LSEC, and efficiency was validated at the mRNA and protein level by qRT-PCR and immunofluorescence, respectively (∗∗p < 0.01, ∗∗∗∗p < 0.0001, Student’s unpaired t test). Scale bars: 100 μm. (B) Flow adhesion assays were performed following PLVAP knockdown (siPLVAP) with Ras-CM-treated LSEC and either monocytes or lymphocytes. Representative phase-contrast images of monocytes are shown. Adhered and transmigrated (% adhered) monocytes and lymphocytes were quantified in 10 visual fields per lane with each condition performed in duplicate. Data shown are mean ± SEM from 3 to 4 independent experiments (∗∗∗p < 0.001, Student’s unpaired t test). Scale bar: 25 μm. (C) Quantification of antibody binding (% area) following treatment of live LSEC with an anti-PLVAP antibody or isotype-matched control (IMC). Cells were then fixed, permeabilized, and stained with an anti-mouse Alexa Fluor 488 secondary antibody. Representative images are shown. Data are mean ± SEM from three independent experiments (∗p < 0.05, Student’s unpaired t test). Scale bars: 20 μm. (D) Flow adhesion assays were performed following antibody-mediated PLVAP blockade with Ras-CM-treated LSEC and either monocytes or lymphocytes. Representative phase-contrast images of monocytes are shown. Adhered and transmigrated (% adhered) monocytes and lymphocytes were quantified in 10 visual fields per lane with each condition performed in duplicate. Data shown are mean ± SEM from three independent experiments (∗p < 0.05, Student’s unpaired t test). Scale bar: 25 μm. (E) Dual immunofluorescent staining of PLVAP (green) and MAC387 (red) in human liver cirrhosis. DAPI (blue) was used as a nuclear counterstain. Scale bars: 100 μm left panel, 20 μm right panel.

Our next aim was to explore the functional contribution of PLVAP to hepatic leukocyte recruitment, as our earlier findings suggested a link between PLVAP, senescence, and immune cell infiltration in CLD (Figure 2). Moreover, PLVAP has previously been implicated as a leukocyte trafficking molecule both *in vitro* and *in vivo*,^12^^,^^13^^,^^14^ thus, we sought to investigate a potential role for PLVAP in SASP-mediated innate/adaptive immune cell recruitment. Flow adhesion assays were performed following PLVAP inhibition in LSEC, either by genetic knockdown or antibody-mediated blockade, and the effects on recruitment of monocytes and lymphocytes were assessed (Figure 5). Transient siRNA transfection of LSEC resulted in robust knockdown of PLVAP mRNA (>95% efficiency) and protein (comparable to IMC) levels, as determined by qRT-PCR and high-content imaging, respectively (Figure 5A). Following small interfering RNA (siRNA) knockdown of PLVAP, we demonstrated that although SASP-mediated monocyte adhesion was unaffected, there was a significant reduction of monocyte transendothelial migration (Figure 5B). This role appeared to be specific for monocytes, because flow adhesion assays with lymphocytes demonstrated no significant effect on adhesion or transmigration, following PLVAP knockdown (Figure 5B).

To validate our PLVAP knockdown findings, we undertook experiments with LSEC following antibody-mediated blockade of PLVAP. Treatment of live LSEC with an anti-PLVAP antibody indicated successful binding within 30 min (Figure 5C). Following antibody-mediated PLVAP blockade, no inhibition of lymphocyte transmigration was observed in response to the SASP. Interestingly, PLVAP inhibition resulted in a small but significant increase in lymphocyte transmigration from 37.3% to 44.1% (Figure 5D). Consistent with siRNA knockdown experiments, monocyte transmigration was selectively impaired following PLVAP antibody treatment, whereas there was also a slight reduction in monocyte adhesion that was not statistically significant (Figure 5D). These data suggest that PLVAP contributes to monocyte, but not lymphocyte, transmigration across liver endothelium in response to the senescent cell secretome. To assess this in the setting of human disease *in situ*, we performed dual-color immunofluorescent staining of PLVAP alongside MAC387, as a marker of infiltrating monocytes, in samples of end-stage CLD. In support of a role for PLVAP in hepatic monocyte recruitment, we were able to spatially demonstrate that MAC387^+^ cells were frequently found adhered to PLVAP^+^ endothelium in CLD patient specimens (Figure 5E).

### PLVAP regulates endothelial paracellular permeability by altering levels of phosphor-VE-cadherin and promoting endothelial gap formation

**Figure 6:**
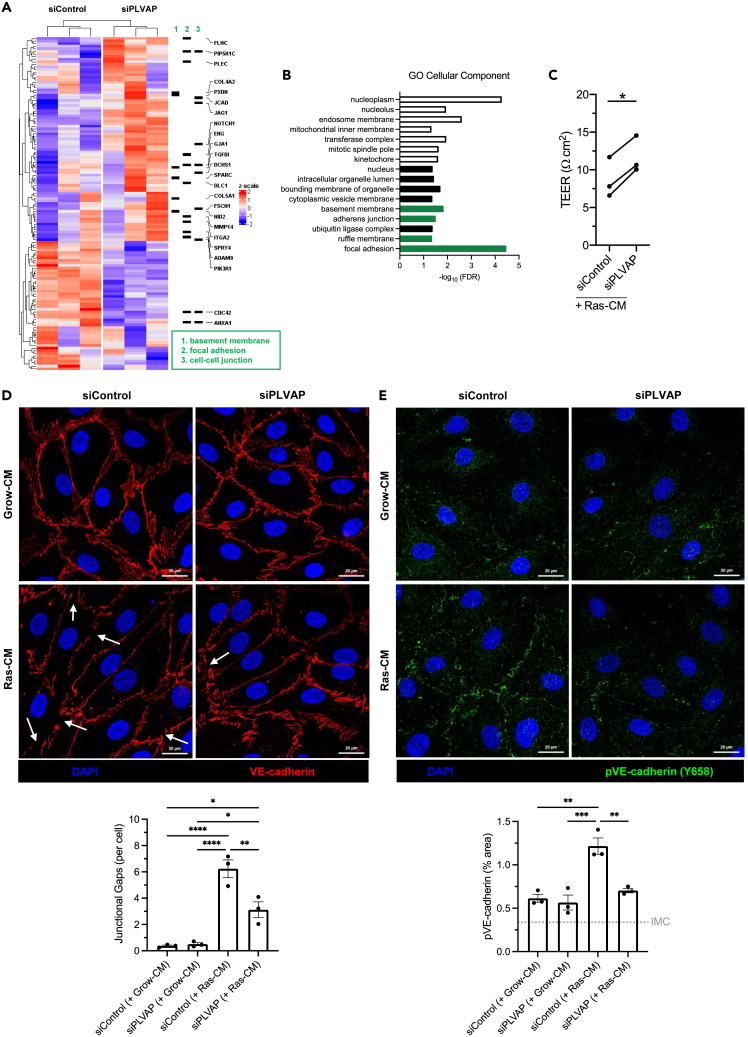
Plasmalemma-vesicle-associated protein (PLVAP) regulates endothelial paracellular permeability by altering intercellular junctions (A) Genetic knockdown of *PLVAP* was performed via siRNA transfection of LSEC (n = 3), and RNA was extracted and subject to bulk RNA sequencing. Heatmap indicates significant differential gene expression, and specific genes from gene ontology pathway analysis are highlighted. (B) Gene ontology (GO) cellular component pathway analysis. Unfilled bars indicate downregulation, and filled bars indicate upregulation in siPLVAP cells. Relevant pathways are highlighted in green. (C) Transendothelial electrical resistance (TEER) of LSEC monolayers following *PLVAP* knockdown (siPLVAP) or negative control (siControl) in the presence (Ras-CM) or absence (Grow-CM) of the senescent secretome. Data shown are mean fold-change to control ±SEM from three independent LSEC isolates (∗p < 0.05, paired t test). (D) Confocal images of VE-cadherin (red) in LSEC following *PLVAP* knockdown and 24 h Ras-CM treatment. DAPI (blue) was used as a nuclear counterstain. Junctional gaps *(lower)* were scored manually for six visual fields per condition and normalized to the cell count. Data shown are mean ± SEM from three independent LSEC isolates (∗p < 0.05, ∗∗∗∗p < 0.0001, one-way ANOVA and Tukey’s post-hoc test). Scale bars: 20 μm. (E) Confocal images of phospho-VE-cadherin (Y658) (green) in LSEC following *PLVAP* knockdown and 24 h Ras-CM treatment. DAPI (blue) was used as a nuclear counterstain. pVE-cadherin % staining area *(lower)* was quantified for six visual fields per condition. Isotype-matched control (IMC) level is indicated by the gray gridline. Data shown are mean ± SEM from three independent LSEC isolates (∗∗p < 0.01, ∗∗∗p < 0.001, one-way ANOVA and Tukey’s post-hoc test).

Unlike CD31, PLVAP did not seem to be enriched around transmigrating monocytes, suggesting its role may be an indirect one (Figure S6A). To elucidate the mechanisms by which PLVAP could regulate monocyte transmigration, bulk RNA sequencing was performed following RNA interference in LSEC. Following *PLVAP* knockdown, 50 genes were significantly downregulated, and 79 genes were significantly upregulated compared with the negative control (*padj* < 0.05) (Figure 6A). Gene ontology analysis revealed an enrichment of several pathways of interest, relating to the basement membrane, focal adhesions, and adherens junctions (Figures 6B and S6B). We demonstrated a significant reduction of transendothelial electrical resistance (TEER), an inverse measurement of permeability, in response to SASP exposure that was reversed upon PLVAP knockdown (Figure 6C). These transcriptional and functional data support a significant role for PLVAP in regulating the barrier function of SASP-stimulated LSEC. We hypothesized that PLVAP regulates junctional permeability, which would explain the downstream effect on monocyte transmigration, given that these cells prefer to extravasate via the paracellular route (Figure 4C).

To test this hypothesis further, we focused on the intercellular adherens junctions by using immunostaining for VE-cadherin (Figure 6D). Following SASP exposure, we noted the formation of spontaneous gaps in LSEC junctions without the presence of any transmigrating cells (Figure 6D), a phenomenon that has been described previously in endothelial cells.^45^ We found that frequency of these junctional gaps per cell was significantly reduced following PLVAP inhibition, suggesting these cells may have tighter adherens junctions in the presence of SASP. VE-cadherin is known to undergo phosphorylation in response to permeability-inducing stimuli such as VEGF, which leads to its internalization from the junction. Immunostaining for phospho-VE-cadherin (Y658) indicated a similar pattern, whereby SASP treatment increased VE-cadherin phosphorylation. In parallel to the reduction in junctional gap formation, we found that SASP-driven phosphor-VE-cadherin upregulation was reversed in the setting of *PLVAP* knockdown (Figure 6E). Therefore, PLVAP has a significant role in regulating LSEC junctional integrity and mediating monocyte transmigration in response to the senescence secretome.

## Discussion

The incidence of CLD continues to increase globally, and for end-stage liver disease and HCC patients, the overall survival remains extremely poor. The outcome of chronic liver injury and/or tumorigenesis is determined, at least in part, by the hepatic immune microenvironment. During chronic inflammation, sinusoidal endothelial cells undergo phenotypic changes that facilitate activation and leukocyte recruitment, which is almost certainly mediated by factors within the biological milieu.^6^ Recent single-cell RNA sequencing studies have highlighted the re-emergence of PLVAP, a marker of fetal liver endothelium largely absent from the adult liver sinusoids, in human liver cirrhosis and HCC.^15^^,^^16^ Here, we validate PLVAP as a marker of scar-associated endothelium within neovessels and peri-fibrotic sinusoidal channels, demonstrating its upregulation in CLD and HCC. Although several soluble mediators of PLVAP expression have been identified in various endothelial cell types, including VEGF,^31^^,^^32^^,^^33^^,^^34^^,^^46^ HGF,^33^ PMA,^33^^,^^35^^,^^47^ TNF-α,^12^^,^^48^ transforming growth factor β,^48^ BMP-9,^49^ angiotensin II,^32^ and fibrinogen,^50^ its specific regulation in primary human liver endothelia has not been studied previously. Given that hepatic endothelial cells are known to differ drastically from conventional endothelia in their transcriptional and metabolic profiles, expression of atypical adhesion molecules, and junctional characteristics,^51^ we sought to investigate PLVAP regulation in primary human LSEC. We found that PLVAP is regulated by several soluble mediators, notably the pro-angiogenic mediator VEGF, as well as conditioned medium from hepatocyte cell lines. Importantly, we also demonstrate a direct link between PLVAP expression and the senescent secretome, to our knowledge a new finding that could make a significant contribution to the upregulation of PLVAP under pathological conditions.

Cellular senescence, a state of proliferative arrest in which cells remain metabolically active, is a key feature of both CLD and HCC.^17^^,^^52^ Yet, the effects of senescence appear to be pleiotropic and are largely cell-type- and context-dependent. For instance, senescence is thought to be a physiological response to cellular stress or damage, which has evolved as a protective mechanism against malignant transformation. However, release of a distinct secretome (SASP) can be both beneficial and deleterious, driving pro- and anti-inflammatory responses.^18^ The senescent cell secretome, a concoction of cytokines, chemokines, and growth factors, has been shown to reinforce senescence in an autocrine and paracrine manner (“bystander effect”), aiming to alert and sensitize neighboring cells to the stressful stimulus.^53^^,^^54^ Furthermore, the SASP activates immune responses to promote senescence surveillance. This surveillance is key in preventing senescent cell accumulation that can simultaneously drive chronic inflammation and foster a pro-tumorigenic niche.^18^^,^^19^ Our results provide evidence for a previously unreported relationship between PLVAP and senescence in CLD, whereby senescent cells are spatially enriched in close association with PLVAP^+^ endothelium in patient specimens.

Endothelia within and proximal to fibrotic regions are known to be major sites for leukocyte recruitment,^55^ and we demonstrate here that peri-fibrotic PLVAP-rich areas were characterized by infiltration of monocytes and lymphocytes. Moreover, we have previously reported that exposure of liver endothelial cells to the senescent cell secretome *in vitro* can drive recruitment of lymphocytes under physiologically low shear stress.^21^ In this study, we demonstrate, using flow adhesion assays and primary human cells, that endothelial SASP stimulation also facilitates the recruitment of monocytes. Furthermore, we provide evidence that SASP-mediated monocyte and lymphocyte recruitment differ on a molecular level, involving distinct transmigratory routes and adhesion molecules. Monocytes are known to transmigrate predominantly via the paracellular route in response to TNF-α stimulation of HUVEC,^56^ and we confirm that this is also the case for SASP-treated LSEC. Similarly, our data suggest ∼60% lymphocytes transmigrate transcellularly, which is consistent with our previous studies using TNF-α-/interferon-γ (IFNγ)-stimulated liver endothelia.^44^ We utilized antibodies targeted against known adhesion molecules to characterize the mechanisms of SASP-mediated leukocyte recruitment. These studies highlighted an important role for CD31 in monocyte transmigration, whereas ICAM1 was implicated in both lymphocyte adhesion and transmigration. Our findings are consistent with previous studies using LSEC and HUVEC, which report the formation of ICAM1-/F-actin-rich adhesive cups and transmigratory channels,^44^^,^^57^ and CD31-/F-actin-rich membrane protrusions,^58^ associated with extravasating lymphocytes and monocytes, respectively.

Among its well-characterized functions in development, vascular permeability, and angiogenesis, PLVAP has also been implicated in leukocyte trafficking both *in vitro* and *in vivo*. In the lymphatic system, PLVAP mediates lymphocyte and antigen entry into the lymph nodes by forming a size-selective sieve,^13^ whereas in the developing liver PLVAP regulates the egress of fetal liver monocytes and subsequent seeding as tissue-resident macrophages.^14^ Yet, a role for PLVAP in adult hepatic leukocyte recruitment has not been studied previously. It has become clear that atypical adhesion molecules, including scavenger receptors, that are enriched in liver endothelia can recruit specific immune cell subsets. Such receptors include stabilin-1 (T_reg_ transmigration) and SCARF1 (CD4^+^ T_eff_ adhesion).^44^^,^^57^ We hypothesized that the disease-specific upregulation of PLVAP within the senescent hepatic microenvironment may be important for leukocyte recruitment. To investigate this, we performed flow adhesion assays with monocytes and lymphocytes following genetic knockdown or antibody-mediated blockade of PLVAP in SASP-treated LSEC. Our data indicate that PLVAP mediates monocyte, but not lymphocyte, transmigration in response to the senescent secretome.

We subsequently explored mechanisms by which PLVAP mediated monocyte transmigration by studying the transcriptional differences between LSEC in the presence and absence of PLVAP knockdown. The gene pathway analysis of our results suggested that PLVAP played a significant role in mediating LSEC barrier function and integrity by regulating the key pathways of focal adhesions, cell-cell junctions, and adherens junctions. We therefore studied the contribution of PLVAP to the barrier function in SASP-treated LSEC and how this could be impacting monocyte transmigration. Firstly, we found that the SASP treatment did increase endothelial permeability with a TEER assay and that this could be rescued by PLVAP knockdown. Subsequently, we linked PLVAP-mediated permeability to junctional changes that would promote paracellular migration by confirming alterations in VE-cadherin gap formation by promoting a shift toward increased phospho-VE-cadherin expression.

LSEC phenotypic changes in human CLD are characterized by loss of fenestrae and the deposition of a basement membrane.^59^ In parallel, there is a re-emergence of the receptor PLVAP, previously shown to be expressed in fetal liver endothelium. Our data provide new insights on how the mechanistic switch in LSEC permeability during the transition from homeostasis to pathology can have an impact on immune cell diapedesis. Considering our data, we propose a mechanism in human CLD in which senescent cell-endothelial crosstalk drives expression of PLVAP and regulates junctional permeability in LSEC to facilitate monocyte recruitment. In the context of cellular senescence, our data provide new insights into the non-cell autonomous impact of the SASP on human liver endothelium. We provide evidence that innate and adaptive immune cell recruitment across liver endothelium occurs by distinct routes, following SASP stimulation, identifying PLVAP as a selective mediator of monocyte transmigration. There is strong evidence that senescence shapes the immune landscape in liver disease, as well as a range of other pathologies, and is an important therapeutic target for chronic inflammation and cancer risk. Despite this, successfully targeting cellular senescence to prevent its deleterious effects while maintaining its beneficial effects remains an unmet clinical need. Our study suggests that directly targeting liver endothelium, specifically PLVAP, could selectively shape the senescence-driven immune microenvironment and have a critical impact on tissue regeneration/cancer risk that accompanies chronic liver inflammation.

### Limitations of the study

There are limitations to our study that need to be taken into account when interpreting the results. Primarily that the functional contribution of PLVAP to monocyte recruitment across liver endothelium has been demonstrated in an *in vitro* setting. We and others have confirmed that PLVAP is upregulated on liver endothelium in cirrhosis,^15^ and our functional analysis was performed on primary human liver endothelial cells. In addition, we have used phase-contrast microscopy and confocal imaging to study transmigration and the route of paracellular and transcellular migration across an endothelial monolayer. Nevertheless, the *in vitro* setting provides limitations with regard to a lack of a multicellular environment, e.g., absence of pericytes, and future *in vivo* studies with intravital microscopy would provide additional support for our work. The links with PLVAP upregulation associated with monocyte recruitment in liver tissue is correlative; future chronic liver injury models that are known to promote senescence could be undertaken in animal models in the setting of PLVAP knockout or inhibition, followed by analysis of monocyte infiltration by imaging and quantification. It is well established that macrophages are highly plastic, and they have diverse roles in liver disease ranging from pathogen recognition to wound healing.^60^ Our studies have focused on the migratory impact of PLVAP on monocyte recruitment, the flow assay recapitulates recruitment within the hepatic sinusoidal channels but we are unable to retrieve transmigrated monocytes. Further *in vitro/in vivo* studies are required to assess if PLVAP has an additional impact on the polarization and phenotype of transmigrated monocytes.

## STAR★Methods

### Key resources table

**Table:** 

REAGENT or RESOURCE	SOURCE	IDENTIFIER
**Antibodies**		

PLVAP	Novus Biologicals	Cat# NBP1-83911; RRID:AB_11029033
Anti-rabbit IgG HRP	Sigma	Cat# A0545; RRID:AB_257896
β-actin	Sigma	Cat# A5441; RRID:AB_476744
Anti-Mouse IgG HRP	Sigma	Cat# A4416; RRID:AB_258167
PLVAP	Abcam	Cat# ab8086; RRID:AB_306255
MR	Abcam	Cat# ab64693; RRID:AB_1523910
L-SIGN	R&D	Cat# MAB162; RRID:AB_2244985
LYVE-1	R&D	Cat# MAB20891; RRID:AB_2297198
CD31	Dako	Cat# M0823; RRID:AB_2114471
CD34	Abcam	Cat# ab8536; RRID:AB_306607
Vimentin	Sigma	Cat# V6389; RRID:AB_609914
PLVAP	Sigma	Cat# HPA002279; RRID:AB_1079636
p21	Dako	Cat# M7202; RRID:AB_2077700
p16	Abcam	Cat# ab54210; RRID:AB_1608104
MAC387	Invitrogen	Cat# MA5-12213; RRID:AB_10981946
CD3	Dako	Cat# M7254; RRID:AB_2631163
CD20	Invitrogen	Cat# MA5-13141; RRID:AB_10983209
NE	Abcam	Cat# ab219585; RRID:AB_11155265
PLVAP	Abcam	Cat# ab81719; RRID:AB_1658370
VE-cadherin	R&D	Cat# MAB9381; RRID:AB_2260374
ICAM-1	R&D	Cat# BBA3; RRID:AB_356950
CCL2	Novus Biologicals	Cat# NBP1-07035; RRID:AB_1625612
GM130	BD Biosciences	Cat# 610823; RRID:AB_398142
CD31	Abcam	Cat# ab9498; RRID:AB_307284
Anti-Mouse Immpress® Kit	Vector	Cat# MP-7402; RRID:AB_2336528
Anti-Rabbit Immpress® Kit	Vector	Cat# MP-7401; RRID:AB_2336529
Anti-mouse IgG1 AF488	Invitrogen™	Cat# A21121; RRID:AB_2535764
Anti-mouse IgG1 AF546	Invitrogen™	Cat# A21123; RRID:AB_2535765
Anti-mouse IgG2a AF546	Invitrogen™	Cat# A21133; RRID:AB_2535772
Anti-mouse mIgG2b AF488	Invitrogen™	Cat# A21141; RRID:AB_2535778
Anti-mouse IgG2b AF546	Invitrogen™	Cat# A21143; RRID:AB_2535779
Anti-rabbit AF488	Invitrogen™	Cat# A11008; RRID:AB_143165
Anti-rabbit AF546	Invitrogen™	Cat# A11035; RRID:AB_2534093
Anti-EpCAM antibody (clone HEA125)	Progen	Cat# 61004 RRID:AB_2920684

**Biological samples**		

Explanted human liver tissues	Queen Elizabeth Hospital, Birmingham, UK.	LREC Approval 06/Q2702/61, 18/WA/0214 and 18/LO/0102, South Birmingham, Birmingham, UK.
Healthy volunteer blood	University of Birmingham, Birmingham, UK	LREC Approval 18/WA/0214, South Birmingham, Birmingham, UK.

**Chemicals, peptides, and recombinant proteins**		

Percoll^TM^	Sigma	Cat# GE17-0891-01
Endothlelial Cell Serum-Free Medium	Gibco	Cat# 11111044
HGF	Peprotech	Cat# 100-39H
VEGF	Peprotech	Cat# 100-21
Collagen type I from rat tail	Sigma	Cat# C3867
Lympholyte® Cell Separation Media	Cedarlane	Cat# CL5020
SiR-actin Live Cell Actin Probe	Spirochrome	Cat# SC001
CellTracker™ Green (CMFDA)	Invitrogen	Cat# C2925
TNFα	Peprotech	Cat# 300-01A
RNAi Max Lipofectamine	Invitrogen	Cat# 13778-075
Direct red 80	Sigma	Cat# 365548
ImmPACT® DAB Peroxidase (HRP) Substrate	Vector	Cat# SK-4105
Picric acid (1.3% v/v)	Sigma	Cat# P6744-1GA
SuperScript® III Reverse Transcriptase	Invitrogen	Cat# 18080-044
TaqMan® Universal PCR Master Mix	Applied Biosystems	Cat# 4326708
SuperScript® III Reverse Transcriptase	Invitrogen	Cat# 18080-044
TaqMan® Universal PCR Master Mix	Applied Biosystems	Cat# 4326708
Phalloidin AF633	Invitrogen	Cat# A22284

**Critical commercial assays**		

Pan Monocyte Isolation Kit (Human)	Miltenyi Biotec	Cat# 130-096-537
RNeasy® Micro Kit	Qiagen	Cat# 74004
RNeasy® Mini Kit	Qiagen	Cat# 74104
Dynabeads™ CD31 Endothelial Cell	ThermoFisher Scientific	Cat# 11155D
Dynabeads™ Goat Anti-Mouse IgG	ThermoFisher Scientific	Cat# 11033
Dynabeads™ CD45	ThermoFisher Scientific	Cat# 11153D
Ibidi® ibiTreat μ-Slide VI 0.4	Ibidi	Cat# 80606

**Deposited data**		

PLVAP siRNA knockdown in human LSEC	Gene Expression Omnibus; https://www.ncbi.nlm.nih.gov/geo/	Gene Expression Omnibus: GSE222993

**Experimental models: Cell lines**		

HepG2	ATCC	Cat# HB-8065; RRID:CVCL_0027
IMR90	ATCC	Cat# CCL-186; RRID:CVCL_0347

**Oligonucleotides**		

Negative Control siRNA	Ambion	Cat# 4390843
PLVAP siRNA	Ambion	Cat# s37972
*18S* TaqMan® Assay	ThermoFisher Scientific	Cat# Hs99999901
*ACTB* TaqMan® Assay	ThermoFisher Scientific	Cat# Hs01060665
*CCL2* TaqMan® Assay	ThermoFisher Scientific	Cat# Hs00234140
*CDKN1A* TaqMan® Assay	ThermoFisher Scientific	Cat# Hs00355782
*CDKN2A* TaqMan® Assay	ThermoFisher Scientific	Cat# Hs00923894
*CXCL8* TaqMan® Assay	ThermoFisher Scientific	Cat# Hs00174103
*GAPDH* TaqMan® Assay	ThermoFisher Scientific	Cat# Hs99999905
*GUSB* TaqMan® Assay	ThermoFisher Scientific	Cat# Hs00939627
*ICAM1* TaqMan® Assay	ThermoFisher Scientific	Cat# Hs00164932
*IL1B* TaqMan® Assay	ThermoFisher Scientific	Cat# Hs00174097
*IL6* TaqMan® Assay	ThermoFisher Scientific	Cat# Hs00985639
*PLVAP* TaqMan® Assay	ThermoFisher Scientific	Cat# Hs00229941

### Resource availability

#### Lead contact

Further information and requests for resources and reagents should be directed to and will be fulfilled by the lead contact, Prof. Shishir Shetty (s.shetty@bham.ac.uk).

#### Materials availability

This study did not generate any new unique reagents.

### Experimental model and study participant details

#### Human tissue and blood

All human tissue was obtained with prior written informed consent and ethically approved for use in research. Explant human liver tissue was collected from patients undergoing liver transplantation at the Queen Elizabeth Hospital Birmingham under ethical study numbers 06/Q2702/61, 18/WA/0214 and 18/LO/0102. Normal liver tissue was obtained from rejected organ donors deemed unsuitable for transplantation under ethical study numbers 06/Q2702/61 and 18/WA/0214. Patient gender/age are provided in Tables S1 and S2. Peripheral blood samples were taken from healthy volunteers under ethical study number 18/WA/0214.

#### Cell lines

HepG2 cells (ATCC) and IMR90 cells (ATCC) were cultured in Dulbecco’s modification of Eagle’s medium (DMEM; GIBCO), supplemented with 10% fetal bovine serum, 100 units/ml penicillin, 100 μg/mL streptomycin and 1% L-glutamine. Cells were maintained in a humidified incubator at 37°C and with 5% CO_2_.

### Method details

#### Quantitative real-time (qRT)-PCR

RNA was isolated from whole liver tissue or cell lysates using the RNeasy Mini Kit (Qiagen) or RNeasy Micro Kit (Qiagen), respectively, as per the manufacturer’s instructions. These kits were used in conjunction with the RNase-Free DNase Set (Qiagen). Approximately 20–30 mg of liver tissue was homogenised in gentleMACS M-tubes (Miltenyi Biotec) containing RLT buffer (RNeasy Kit) using a gentleMACS Dissociator (Miltenyi Biotec). Alternatively, primary liver cells were cultured in 6-well TC-treated plates (Corning) and lysed *in situ* with RLT buffer containing 1% β-mercaptoethanol. Following RNA extraction, quantity and purity were assessed using a NanoPhotometer (Geneflow), and reverse transcription was performed using the SuperScript III Reverse Transcriptase Kit (ThermoFisher Scientific). qRT-PCR was performed in triplicate to assess mRNA expression using TaqMan Gene Expression Assays (Applied Biosystems) and TaqMan Universal PCR Master Mix. Reactions were performed in a LightCycler 480 (Roche) by completing 40 cycles of the following program: 95°C for 10 s; 60°C for 50 s; 72°C for 1 s. Gene expression was normalised to an appropriate housekeeping gene (*GAPDH* for cells, *18S* for CLD tissue, *GUSB* for HCC tissue) using “E-analysis” software (Roche).

#### Western blot

Approximately 75 mg of frozen liver tissues were homogenised in gentleMACS M-tubes (Miltenyi Biotec) containing CelLytic MT lysis buffer (Sigma), 0.1% Protease Inhibitor Cocktail (Sigma), 1% Phosphatase Inhibitor Cocktail 3 (Sigma) and 5 U/mL DNase-I (Sigma). Protein concentration was determined by bicinchoninic acid (BCA) assay (Sigma), using bovine serum albumin (BSA) as a protein standard, and subsequently diluted to a concentration of 2 mg/mL in CelLytic MT lysis buffer before storage at −20°C. Protein lysates (20 μg) were separated on a 10% SDS-PAGE and transferred to nitrocellulose membranes (ThermoFisher Scientific) before blocking with 5% non-fat milk solution (Marvel) in PBS +0.02% Tween 20 (Sigma) (PBS/T) for 1 h at 20°C–22°C. Primary antibody was incubated overnight at 4°C before washing three times with PBS/T followed by addition of a horseradish peroxidase (HRP)-conjugated anti-rabbit IgG antibody and incubation for 1 h at 20°C–22°C. Following three additional wash steps with PBS/T, protein bands were detected using Pierce Enhanced Chemiluminescence (ECL) Substrate (ThermoFisher Scientific). Membranes were stripped with Restore Western Stripping Buffer (ThermoFisher Scientific) for 10 min before repeating blocking and incubation steps above to probe for β-actin. After washing, membranes were incubated with HRP-conjugated anti-mouse IgG antibody for 1 h. Protein bands were detected as described above.

#### Immunohistochemical, immunofluorescent and histological tissue staining

Immunohistochemistry was performed on 7 μm-thick acetone-fixed cryo-sections and 3 μm-thick formalin-fixed paraffin-embedded (FFPE) sections. Frozen sections were thawed to 20°C–22°C and hydrated with PBS/T (0.1%) for 5 min. FFPE sections were de-waxed and rehydrated by static sequential incubation for 3 min in xylene (3x), industrial denatured alcohol (3x), and water (2x). Heat-induced epitope retrieval was performed for FFPE sections for 20–30 min using 1% Tris-based (pH 9) or Citrate-based (pH 6) Antigen Retrieval Buffer (Vector Laboratories) followed by washing with TBS/T. Endogenous peroxidase activity was blocked using Bloxall Endogenous Blocking Solution (Vector Laboratories) for 15 min, followed by one PBS/T or TBS/T wash. To block non-specific antibody binding, sections were incubated with 2X casein solution (Vector Laboratories) in PBS/TBS for 20 min at 20°C–22°C. For immunofluorescent (IF) staining of cryo-sections, blocking of non-specific binding was performed immediately after thawing and rehydration, with 2X casein containing 10% goat serum in PBS, for 20 min at 20°C–22°C.

Primary antibodies were incubated for 1 h at 20°C–22°C, or overnight at 4°C (p21), before two 5-min PBS/T or TBS/T washes. Isotype-matched controls at equivalent concentrations were performed for each batch of staining. Sections were then incubated with an appropriate HRP-conjugated secondary antibody (Anti-Mouse [#MP-7402] or Anti-Rabbit [#MP-7401] Immpress Kit, Vector Laboratories), or fluorescently-conjugated secondary antibody (Invitrogen), for 30 min at 20°C–22°C before two additional 5-min wash steps with PBS/T or TBS/T. Chromogenic staining was visualised by incubating with ImmPACT DAB Peroxidase (HRP) Substrate (Vector Laboratories #SK-4105) for 5 min followed by PBS/T or TBS/T wash. For IF staining, DAPI was used as a nuclear counterstain (300 nM in distilled water) by incubating with sections for 5 min followed by washing with PBS/T or TBS/T. Autofluorescence of FFPE sections was quenched using TrueView Autofluorescence Quenching Kit (Vector Laboratories) as per the manufacturer’s instructions. Sections were then mounted using VECTASHIELD Vibrance Antifade Mounting Medium (IHC-P) or ProLong Gold Antifade Mountant (IHC-Fr).

Sirius Red staining was performed on matched serial liver sections, where possible, by incubating sections with 5% phorbol 12-myristate 13-acetate (PMA) in dH_2_O for 5 min, followed by incubation for 30–60 min with Sirius Red solution (1.3% picric acid containing 1 g/L Direct Red 80, Sigma). Sirius Red was then briefly replaced with 0.1 M hydrochloric acid before rinsing with distilled water. For chromogenic and Sirius Red staining, Mayer’s Haematoxylin (pfm Medical) was used as a nuclear counterstain by incubating with sections for 1 min before washing in warm water for 2 min. Stained sections were then cleared and dehydrated in industrial denatured alcohol (3x) and xylene (3x) before being mounted with DPX (Phthalate-free) mounting medium (CellPath).

All stained sections were imaged using an Axio Scan.Z1 slide scanner and visual fields were selected post-acquisition using ZEN blue software (Zeiss). To quantify immunohistochemical staining, the “Threshold” and “Measure” functions of ImageJ software were used to give a mean % positive staining area for five visual fields, allowing comparison of expression between liver samples.

#### Primary cell isolation and culture

All cells were cultured at 37°C in a humidified incubator with 5% CO_2_ in tissue culture flasks (Corning). Following isolation, all cells were passaged using TrypLE Express Enzyme (1X) (Gibco). Primary LSEC, activated liver myofibroblasts (aLMF), quiescent hepatic stellate cells (HSC) and biliary epithelial cells (BEC) were isolated from ∼30 g slices of explanted human liver as previously described.^30^^,^^61^^,^^62^ Briefly, tissue was mechanically and enzymatically digested (10 mg/mL collagenase in PBS, Sigma), before separation of the non-parenchymal cell fraction by centrifugation (800 *g* for 20 min) on a 33%:77% Percoll (GE Healthcare) density gradient. Immune cells were depleted by positive immunomagnetic selection using CD45-conjugated Dynabeads (Invitrogen). BEC were isolated by positive immunomagnetic selection for epithelial cell adhesion molecule (EpCAM, Progen #61004, 4.55 μg/mL) using Goat Anti-Mouse IgG Dynabeads (Invitrogen). BEC were maintained in Ham’s F12 Medium and DMEM (1:1) supplemented with 10% human serum (TCS Biosciences), 1% penicillin-streptomycin-glutamine (PSG) (Gibco), 10 ng/mL epidermal growth factor (EGF, Peprotech), 10 ng/mL hepatocyte growth factor (HGF, Peprotech), 0.124 IU/mL insulin, 2 μg/mL hydrocortisone, 10 ng/mL cholera toxin (Sigma) and 2 nM tri-ido-thyronine (Sigma). Endothelial cells were isolated by positive immunomagnetic selection using CD31 antibody-conjugated Dynabeads (Invitrogen) and seeded in rat tail collagen (RTC)-coated (40 μg/mL) tissue culture flasks. LSEC were maintained in Human Endothelial Serum-free Medium (Gibco) supplemented with 10% human serum, 1% PSG, 10 ng/mL VEGF (Peprotech) and 10 ng/mL HGF. Whilst some phenotypic loss of LSEC *in vitro* will occur, we have previously demonstrated that the primary human LSEC isolated from diseased liver tissue using our technique still maintain their critical phenotypic features of scavenger receptor expression and highly efficient endocytosis and maintain a distinct gene signature compared to conventional endothelium, human umbilical vein endothelial cells, up to passage 5(51). Endothelial cells were used up to passage 5 except in flow assays where only passage 4 cells were used. Following removal of BEC and endothelial cells, remaining cells were considered to be a heterogeneous population of aLMFs, which were cultured in Dulbecco’s Modified Eagle Medium (DMEM) (Gibco) supplemented with 16% fetal bovine serum (FBS) (Gibco) and 1% PSG. Quiescent HSCs were isolated only from non-fibrotic liver tissue, cultured in DMEM containing 16% FBS and 1% PSG, and used up to passage 4.

#### Immunocytochemistry

Endothelial cells were seeded in RTC-coated ibiTreat μ-slide VI 0.4 (Ibidi) and grown to confluence before being fixed with 4% paraformaldehyde for 10–15 min at 37°C. All remaining incubation steps were performed at 20°C–22°C with gentle agitation. Cells were permeabilised by incubating with 0.3% Triton X-100 in PBS for 5 min, before blocking with PBS containing 2X casein buffer and 10% goat serum for 20 min. Primary antibodies were then diluted in PBS and incubated for 1 h followed by three PBS/T (0.1%) washes. Cells were incubated with fluorescently-conjugated secondary antibodies for 30 min before three additional wash steps. DAPI (300 nM in dH_2_O) was used as a nuclear counterstain and incubated for 5 min before one PBS/T wash followed by addition of PBS. For relevant experiments, Phalloidin Alexa Fluor 633 (Invitrogen) was used to stain F-actin. Stained cells were stored at 4°C prior to imaging on a LSM880 confocal microscope (Zeiss).

#### High-content imaging

Primary human liver sinusoidal endothelial cells (LSEC) were seeded in RTC-coated black 96-well imaging plates (Falcon) and grown to confluence. Cells were then serum- and growth factor-starved for 2 h before 24-h treatment with 10 ng/mL tumor necrosis factor α (TNFα), 100 ng/mL VEGF, conditioned medium from HepG2 hepatoma cells, or a relevant vehicle control. HepG2 cells were cultured in DMEM supplemented with 10% FBS and 1% PSG. Prior to harvesting of supernatants, HepG2 cells were grown to approximately 80% confluence, before passaging into two flasks and taking conditioned medium 24 h after splitting. Supernatants were centrifuged at 300 *g* for 5 min to remove cell debris and stored at −20°C prior to use in high-content imaging assays. Following treatment, LSEC were fixed with 4% PFA for 10–15 min and PLVAP was visualised by immunocytochemistry as described above. Images were acquired on a CellInsight CX5 High Content Screening Platform (ThermoFisher Scientific) and immunofluorescence was analyzed using integrated HCS Studio Cell Analysis Software. A spot detection algorithm was applied to detect signal, on a per cell basis, within a defined region of interest relative to a primary object (cell nucleus). “Object.SpotTotalArea.Ch2” and “Object.SpotTotalInten.Ch2” were chosen as the most appropriate parameters to give fluorescence area and intensity measurements, respectively (Figure S2).

#### SASP generation and stimulation

The SASP was generated by obtaining conditioned medium from IMR90 cells, expressing a 4-hydroxytamoxifen (4-OHT)-inducible form of oncogenic HRAS^G12V^ (ER:HRAS^G12V^), undergoing oncogene-induced senescence.^21^ ER:HRAS^G12V^ IMR90 cells were generated using the pLNCX2 ER:ras (Addgene #67844; RRID:Addgene_67844) retroviral vector and maintained in DMEM supplemented with 10% FBS and cultured at 37°C in 5% CO_2_. Cells were cultured in the presence or absence of 100 nM 4-OHT (Sigma) and conditioned medium was harvested on day 6, centrifuged at 300 *g* for 5 min, and stored at −80°C until use. The SASP was designated “Ras-CM” and the growing control was designated “Grow-CM”. LSEC were treated with either Ras-CM or Grow-CM diluted in endothelial medium (1:1 or 1:3 ratio) for 24 h before downstream analysis (i.e., qRT-PCR, immunocytochemistry, flow adhesion assays).

#### RNA interference

LSEC were seeded in RTC-coated 6-well or 96-well plates and analysis of knockdown efficiency at the mRNA and protein level was performed by qRT-PCR and high-content imaging, respectively. For use in flow adhesion assays, LSEC were seeded in RTC-coated ibidi μ-slides and siRNA knockdown was performed *in situ.* siRNA duplexes (PLVAP, Ambion #s37972; negative control, Ambion #4390843) were diluted in Opti-MEM Reduced Serum Medium (Gibco), such that the final concentration was 3.125 nM, and incubated at 20°C–22°C for 10 min. RNAi Max Lipofectamine (Invitrogen) was diluted in Opti-MEM to a final concentration of 0.3% and incubated for 10 min at 20°C–22°C. Lipofectamine and siRNA solutions were then mixed and incubated for a further 10 min at 20°C–22°C, before addition of Opti-MEM to a final volume of 1 mL/well for 6-well plate and 200 μL/well for 96-well plates and ibidi μ-slides. Cells were washed twice with PBS and then siRNA/lipofectamine solution was incubated with LSEC at 37°C for 4 h. This was then replaced with antibiotic- and growth factor-free culture medium and incubated for 48 h, prior to cell lysis, fixation, or use in flow adhesion assays.

#### Leukocyte isolation

Peripheral blood mononuclear cells (PBMCs) were isolated from whole blood using Lympholyte cell separation medium (Cedarlane) and centrifugation at 800*g* for 20 min with no brake. To isolate lymphocytes, PBMCs were resuspended in flow assay medium (Endothelial SFM +0.1% bovine serum albumin) and placed in a TC-treated culture flask for 1 h to allow monocyte adhesion. Floating cell suspension was then harvested, centrifuged, and resuspended in flow assay medium at a cell density of 1 x 10^6^/mL. Monocytes were isolated from the PBMC fraction by negative immunomagnetic selection using a Pan Monocyte Isolation Kit (Miltenyi Biotec) according to the manufacturer’s instructions. Briefly, PBMCs were incubated for 5 min on ice with Fc Receptor Blocking Reagent and Biotin-Antibody Cocktail diluted in ice-cold magnetic-associated cell sorting (MACS) buffer (PBS +1 mM ethylenediaminetetraacetic acid (EDTA) and 2% FBS). Then, Anti-Biotin Microbeads were added and further incubated on ice for 10 min. Labeled cell suspension was then added to a pre-wetted LS column (Miltenyi Biotec) fitted within a Magnet Midi MACS (Miltenyi Biotec), followed by three MACS buffer washes (3 mL) and collection of the flow-through. Monocytes were kept in MACS buffer until required and then resuspended in flow assay medium at a cell density of 1 x 10^6^/mL.

#### Flow adhesion assay

Flow adhesion assays were performed to recapitulate leukocyte recruitment within the hepatic sinusoids as previously described.^42^ Approximately 75,000 LSEC per channel were seeded into RTC-coated ibidi μ-slides and allowed to form an endothelial monolayer overnight. LSEC were stimulated for 24 h prior to flow assay with either Grow-CM or Ras-CM diluted in Endothelial SFM (1:1 for lymphocytes, 1:3 for monocytes). All flow assay conditions were performed in duplicate. For siRNA knockdown experiments, flow assays were performed on day four (48 h post-knockdown), whilst all other flow assays were performed on day three. For antibody-blockade experiments, LSEC were treated with 10 μg/mL relevant antibody or isotype-matched control (IMC) for 40 min, and flow assays were performed immediately thereafter. Leukocytes were perfused over LSEC at a physiological shear of 0.05 Pa for 5 min/channel, at a density of 1 x 10^6^/mL, followed by a 3-min wash with flow assay medium. Phase-contrast recordings were taken against the flow direction. The use of a phase contrast microscope allows clear delineation of leukocytes adherent to the endothelial layer but remaining on the luminal side (phase bright in appearance) and those that have performed diapedesis and are below the endothelial layer (phase dark), as previously described.^42^ Leukocyte adhesion, shape-change and transmigration were scored manually using ImageJ for ten visual fields/channel. Mean cell counts per channel were then normalised to cells/mm^2^/10^6^ cells perfused using the following equation:

$$N=\frac{c}{r\times b\times a\times l}$$

where N is the normalised count, c is the cell count per visual field, r is the flow rate (0.28 mL/min), b is the bolus time (5 min), a is the visual field area (0.154 mm^2^) and l is the leukocyte density (1 x 10^6^/mL)

The proportion of cells undergoing activation (shapechange) and transendothelial migration are expressed as a % of the total adherent cells.

#### Analysis of leukocyte Rolling, adhesion and transmigration

Recorded fields of view were analyzed offline and the number of adhered, shape-changed and transmigrated cells were scored manually by eye using the “Cell Counting” plugin on ImageJ. Stable, round, phase-bright leukocytes were counted as “adhered”, whilst leukocytes that were phase-dark were considered “transmigrated”. Shape-changed cells were no longer round and could be partially phase-bright/phase-dark.

To characterise the route of leukocyte transmigration in response to the senescent secretome, flow adhesion assays were performed with fluorescently-labelled (CellTracker Green and SiR-actin) Ras-CM-treated LSEC and peripheral blood monocytes or lymphocytes. Transmigration events were first identified by the presence of a leukocyte (visualised with DAPI and distinguished from LSEC nuclei based on size), along with disruption of the LSEC cytoplasm (CellTracker Green), which allowed differentiation between leukocytes adhered to the LSEC monolayer and those protruding through the LSEC cell body. Route of was transmigration was first determined by the location of diapedesis, i.e., at the cellular junction or not, and then by the following: 1) transcellular transmigration was further identified by the presence of an actin-rich ‘ring’ around the leukocyte, visualised by pre-labelling the LSEC cytoskeleton with the live cell actin probe, SiR-actin. 2) paracellular transmigration was further identified by a definitive break in the integrity of VE-cadherin staining at the cell junctions.

#### Transendothelial electrical resistance (TEER) assay

Approximately 150,000 LSEC were seeded into RTC-coated 24-well Millicell transwell inserts (polyethylene terephthalate, 0.4 μm; EMD Millipore) and allowed to form an endothelial monolayer overnight. siRNA knockdown was performed *in situ* and LSEC were then incubated in antibiotic- and growth factor-free culture medium for 24 h. LSEC were stimulated for a further 24 h, prior to the TEER assay, with Ras-CM diluted in Endothelial SFM (1:3). All conditions were performed in duplicate. Transendothelial electrical resistance (TEER) was measured using a Millicell-ERS2 Volt-Ohm Meter (EMD Millipore) and expressed in Ωcm2.

#### RNA sequencing

Paired end 2x150 bp RNA sequencing was performed by Source BioScience and we received raw sequencing files after adapter trimming. Transcript expression was quantified using the selective-alignment mapping algorithm implemented in *Salmon* (v1.5.2)^63^ using a decoy-aware transcriptome index built from the reference genome GRCh38.p13 and the Gencode version 38 transcriptome. *Salmon* quantification was run with *--validateMappings*, *--gcBias* and *--seqBias* options and otherwise default settings. Gene-wise count summarisation and data import into R version 4.1.2 was performed with *tximport*.^64^ Differential gene expression analysis was done using *DESeq2* (v1.34.0) with *independentFiltering* set to false and otherwise default settings.^65^ Batch effects between LSEC samples from different livers were accounted for by including the liver sample identity into the design formula. Effect sizes were adjusted by Bayesian shrinkage of log_2_-fold changes as implemented in the *apeglm* package.^66^ Functional enrichment analysis was performed on differentially expressed genes by PANTHER overrepresentation Fisher’s Exact test and log_10_ false discovery rates were calculated.

### Quantification and statistical analysis

All statistical analysis was performed using Prism 9.1.0 software (GraphPad). All data were tested for normal (Guassian) distribution using a Shapiro-Wilk normality test. Data presented graphically show mean ± standard error of the mean (SEM) (parametric) or median ± interquartile range (IQR) (non-parametric) unless otherwise indicated in results. Two independent datasets were compared by student’s unpaired *t*-test (parametric) or Mann-Whitney test (non-parametric). Paired data were compared using a student’s paired *t*-test (parametric) or Wilcoxon matched-pairs signed rank test (non-parametric). Where appropriate, correlation analysis was performed for parametric or non-parametric data using a Pearson’s or Spearman’s correlation test, respectively. A p value of <0.05 was considered statistically significant.

## Data Availability

• RNA-sequencing data are publicly available through the Gene Expression Omnibus: GSE222993. • This paper does not report any original code. • Any additional information required to reanalyse the data reported in this paper is available from the lead contact upon request.
