# Supplementary material for: The senescent secretome drives PLVAP expression in cultured human hepatic endothelial cells to promote monocyte transmigration
Source: iScience. 2023 Sep 19;26(10):107966. doi: 10.1016/j.isci.2023.107966 (PMC10558774; doi:10.1016/j.isci.2023.107966)
Supplement: Document S1. Figures S1–S6 and Tables S1 and S2 [file mmc1.pdf]

iScience, Volume *26*

Supplemental information

The senescent secretome drives PLVAP expression in cultured human hepatic endothelial cells to promote monocyte transmigration

Alex L. Wilkinson, Samuel Hulme, James I. Kennedy, Emily R. Mann, Paul Horn, Emma L. Shepherd, Kelvin Yin, Marco Y.W. Zaki, Gareth Hardisty, Wei-Yu Lu, Pia Rantakari, David H. Adams, Marko Salmi, Matthew Hoare, Daniel A. Patten, and Shishir Shetty

Supplementary Figure 1

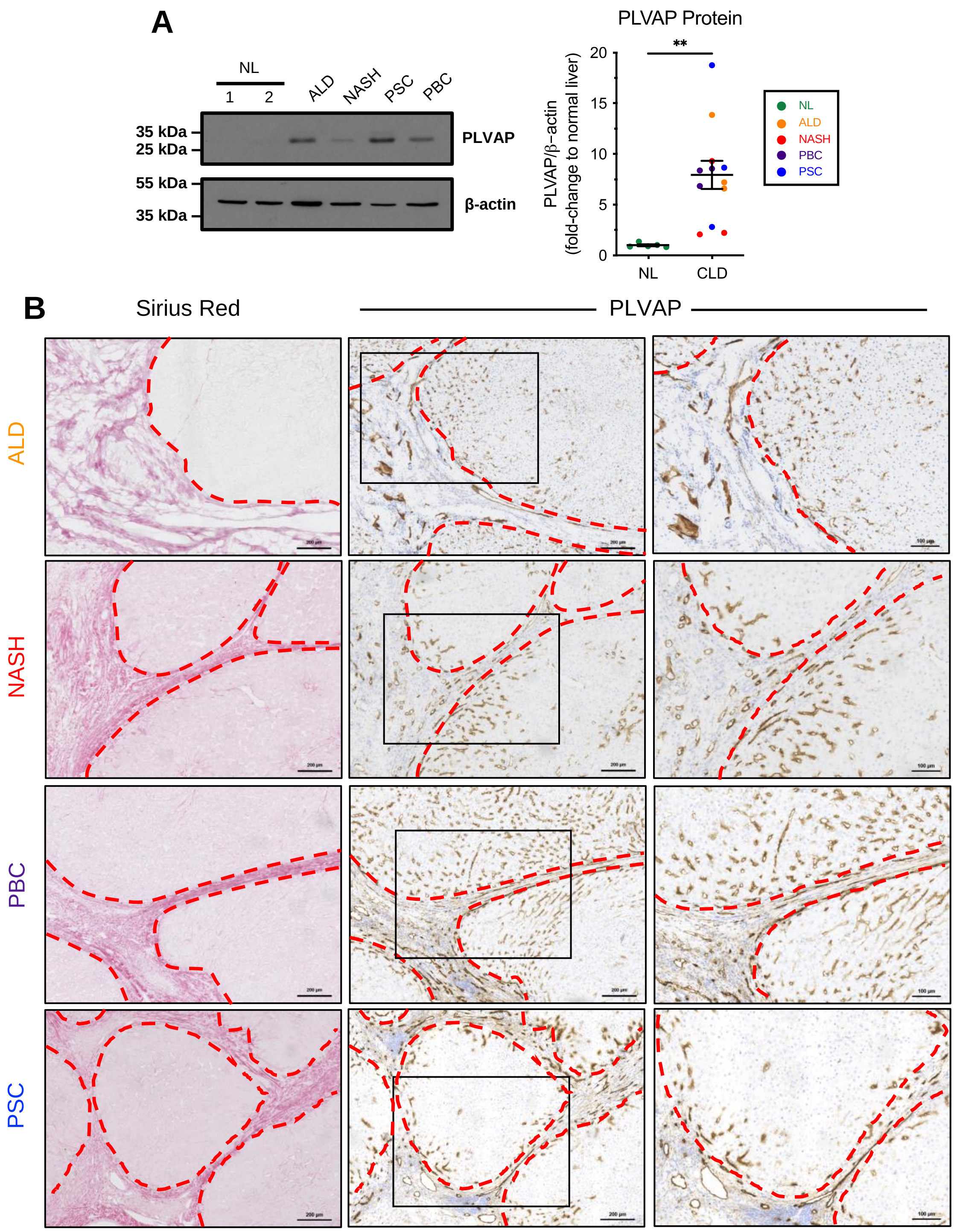

Supplementary Figure 1, Related to Figure 1

Plasmalemma vesicle-associated protein (PLVAP) protein expression is upregulated across multiple human liver aetiologies.

(A) Western blot (*left*) and quantification (*right*) showing PLVAP protein expression (relative to β-actin) in normal (n=5) vs. cirrhotic (n=12) livers. Data are normalised to fold-change to NL and are mean ± SEM (**p<0.01, student's t-test)

(B) Representative IHC images of PLVAP and Sirius Red in matched serial sections from each liver aetiology. Fibrotic septa are indicated by red dashed lines. NASH images are those shown in Figure 1D

Supplementary Figure 2

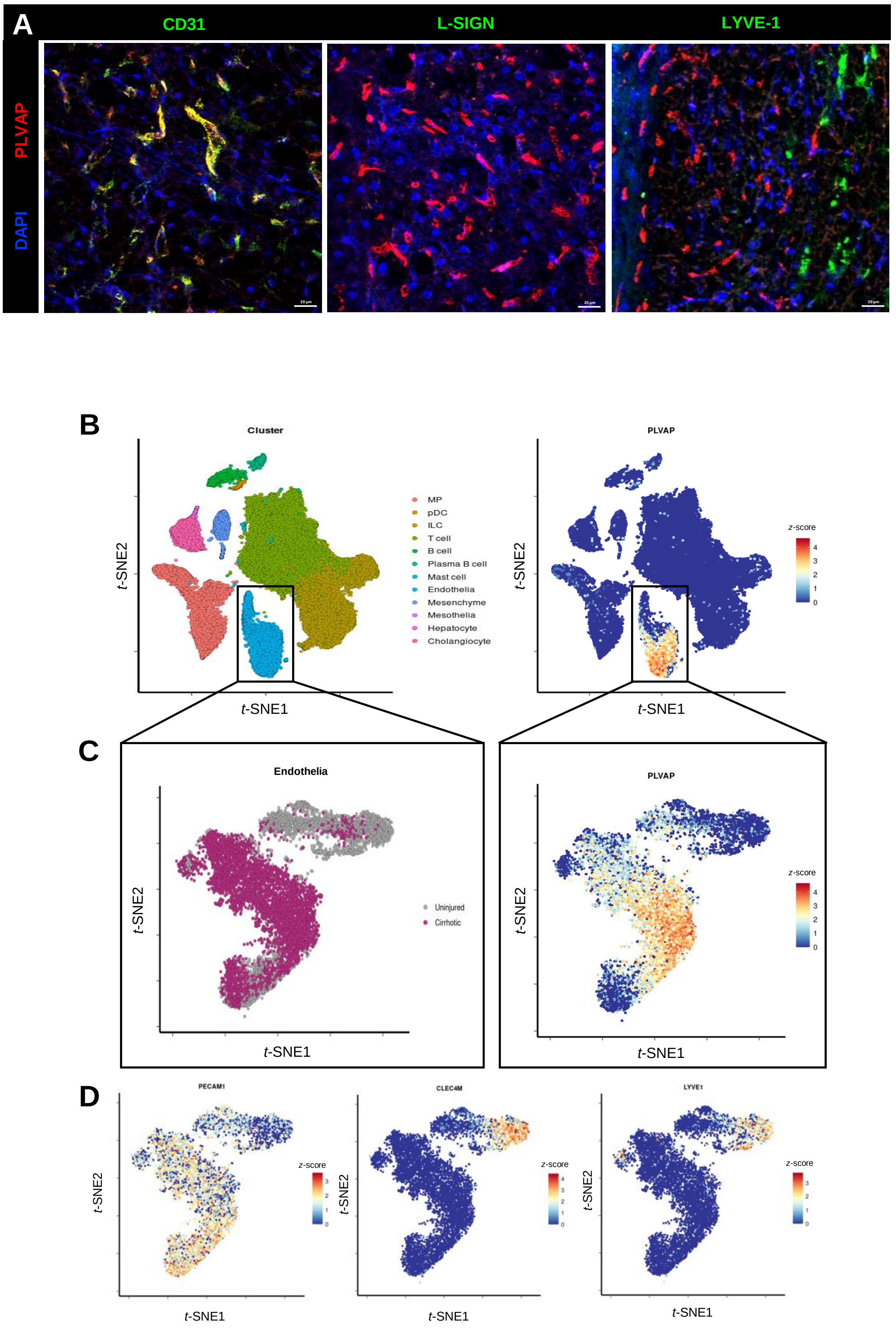

Supplementary Figure 2, Related to Figure 1

***PLVAP* is enriched in scar-associated endothelial cells from cirrhotic patients.**

(A) Representative high magnification images from chronic liver disease demonstrating colocalization with CD31+ endothelial cells within the hepatic sinusoids.

(B) Publicly-available RNA sequencing data in normal (n=5) and cirrhotic (n=5) human livers. Cell lineage was inferred from expression of marker gene signatures (*left*). *PLVAP* expression in all lineages is shown (*right*). All data is accessible via https://shiny.igmm.ed.ac.uk/livercellatlas/

(C) Clustering of endothelial cells from normal (n=4) and cirrhotic (n=3) human liver (8020 cells in total) with injury condition annotated (*left*). *PLVAP* expression in cells of endothelial lineage (*right*)

(D) Single cell data confirming co-expression of PLVAP with PECAM1 (CD31) endothelial cells in cirrhotic livers and absence of PLVAP from CLEC4M (L-SIGN) and LYVE-1 expressing endothelial populations.

Supplementary Figure 3

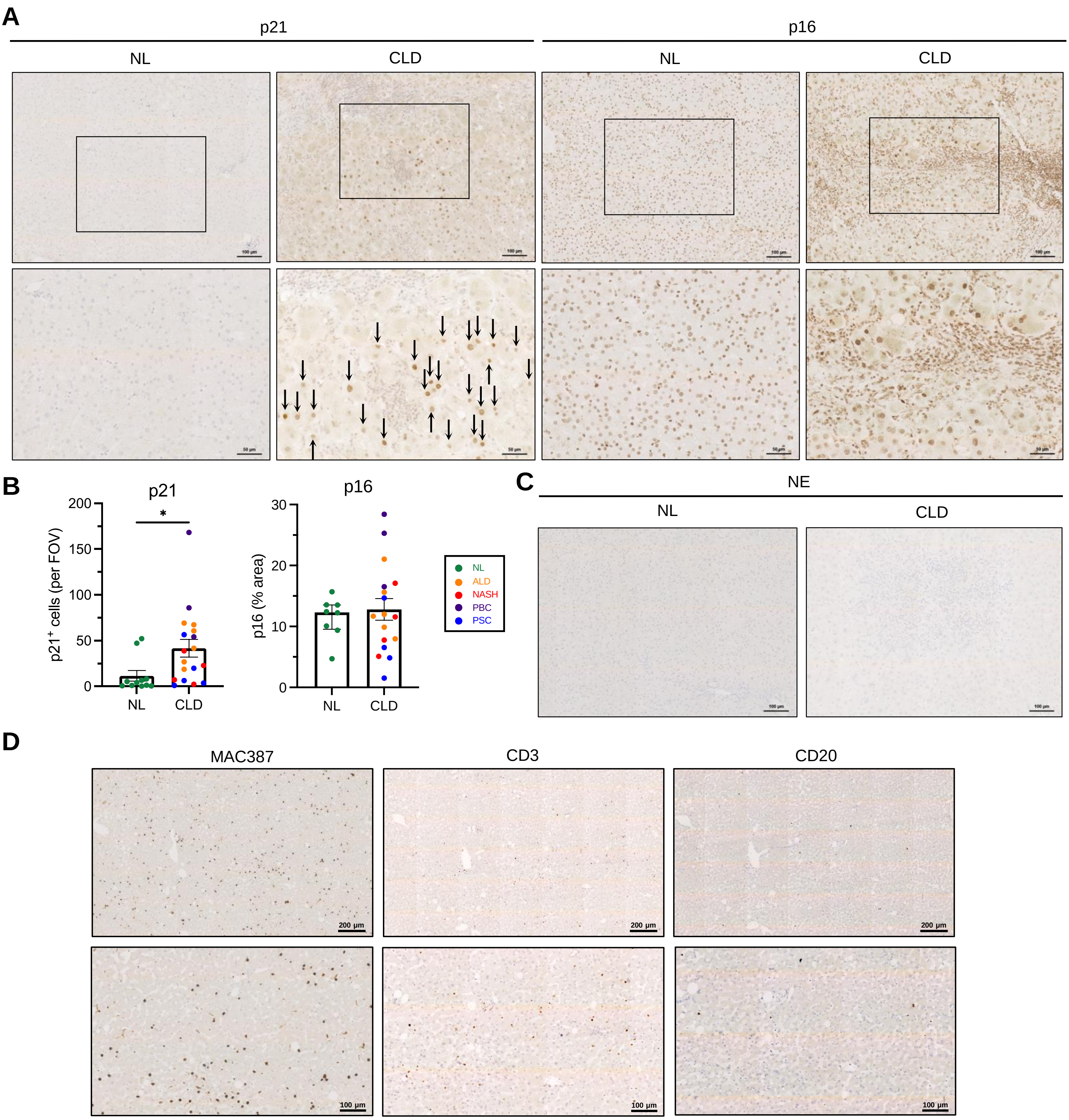

Supplementary Figure 3, Related to Figure 2

Expression of senescence and immune cell markers in normal liver (NL) and chronic liver disease (CLD)

(A) Representative low (*upper*) and high (inset, *lower*) power images of p21 and p16 immunohistochemical staining in NL and CLD

(B) Quantification of immunohistochemical staining for p21 (positive cells per field of view (FOV)) (*left*) or p16 (% area) (*right*) in NL compared to CLD. Data shown are mean ± SEM (*$p<0.05$, student's unpaired t-test)

(C) Representative immunohistochemical staining of neutrophil elastase (NE) in NL and CLD. Visual fields shown for NL and CLD are the same as those in (D) and Figure 2D, respectively

(D) Representative low (*upper*) and high (*lower*) power images of immune markers, MAC387, CD3 and CD20 (*from left to right*), in serial sections from NL

Supplementary Figure 4

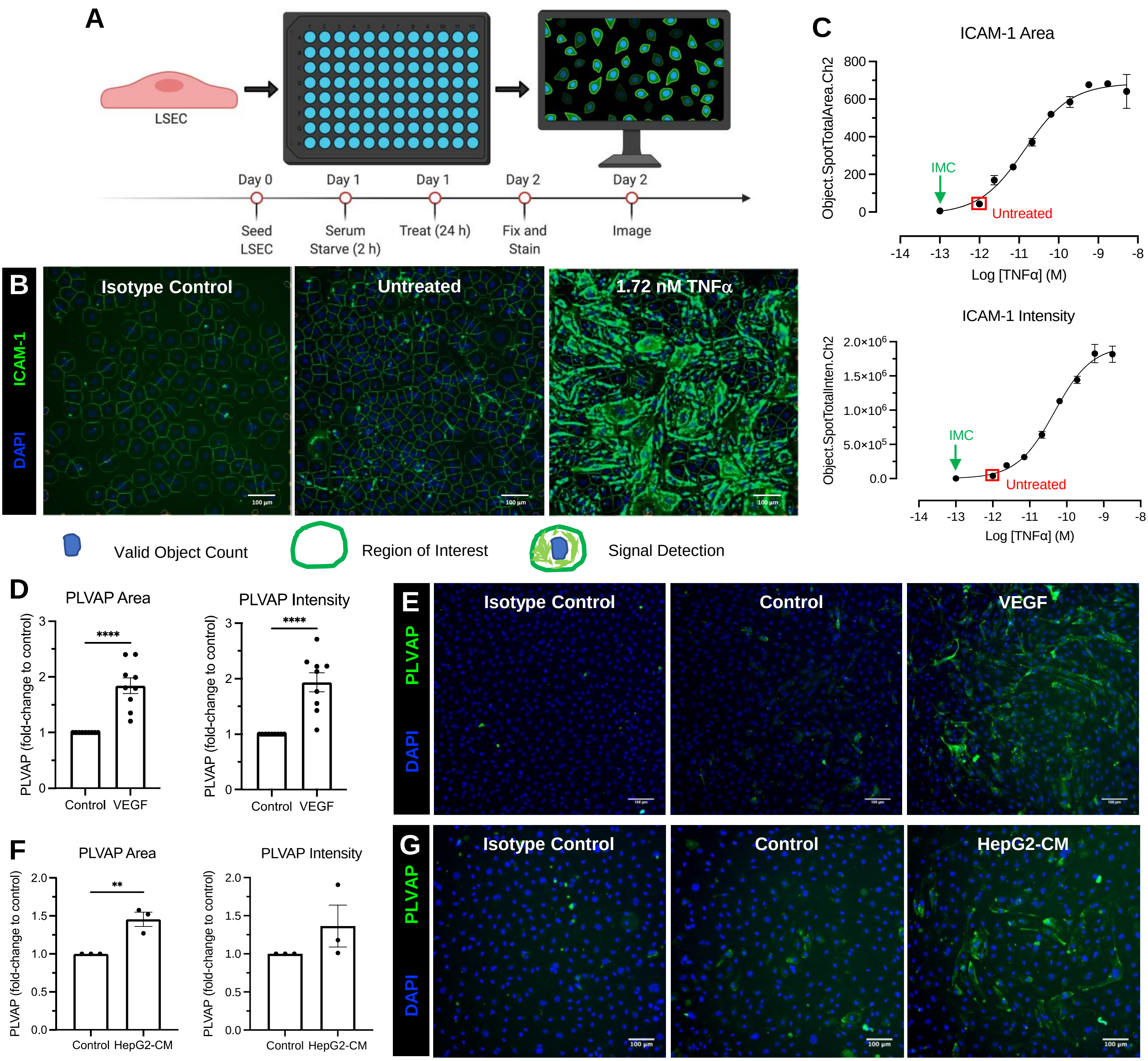

Supplementary Figure 4, Related to Figure 3

High-content imaging of liver sinusoidal endothelial cells (LSEC) to study regulation of plasmalemma vesicle-associated protein (PLVAP) *in vitro*

(A) High-content imaging assay protocol to study regulation of PLVAP in human primary LSEC *in vitro*

(B) High-content image analysis was optimised and validated by staining for ICAM-1 (green) in untreated and TNFα-stimulated LSEC. DAPI (blue) was used as a nuclear counterstain

(C) Dose-response curves for ICAM-1 fluorescence area (*upper*) and intensity (*lower*) following 24 h stimulation with increasing concentrations of TNFα. Data shown are mean ± SD from one cell isolate with each condition performed in duplicate

(D) PLVAP area (*left*) and intensity (*right*) following 24 h treatment with VEGF (100 ng/mL) (n=9). Data shown are mean ± SEM (****p<0.0001, student's unpaired *t*-test) with each condition performed in at least duplicate

(E) Representative high-content images of PLVAP immunofluorescence (green), including isotype control, for data shown in (D)

(F) PLVAP area (*left*) and intensity (*right*) following 24 h treatment with conditioned medium from HepG2 hepatoma cells (HepG2-CM) (n=3). Data shown are mean ± SEM (**p<0.01, student's unpaired *t*-test) with each condition performed in at least duplicate

(G) Representative high-content images of PLVAP immunofluorescence (green), including isotype control, for data shown in (F)

Supplementary Figure 5

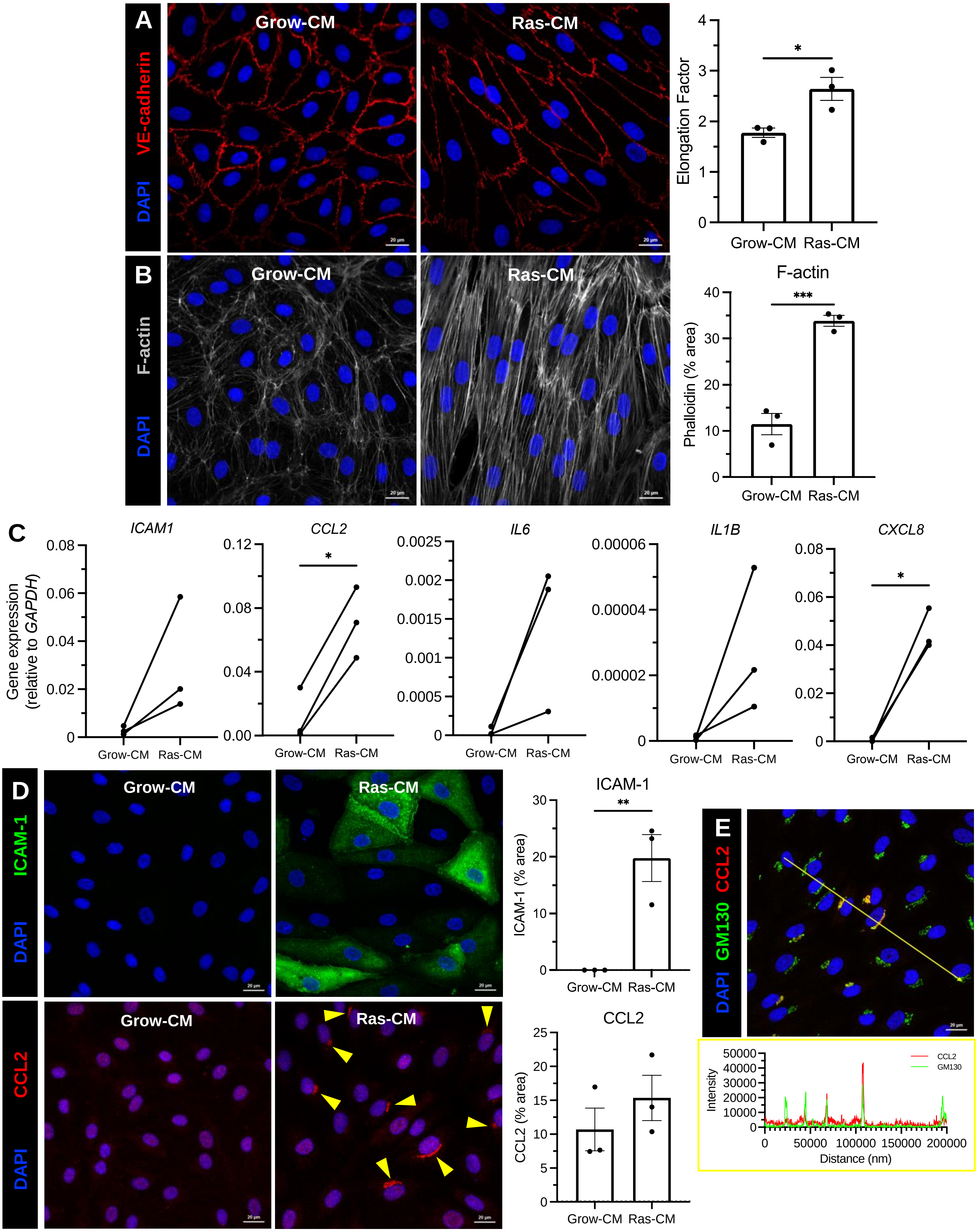

Supplementary Figure 5, Related to Figure 4
The senescent secretome activates primary human liver sinusoidal endothelial cells (LSEC) and upregulates expression of proinflammatory cytokines, chemokines and adhesion molecules

(A) Morphological changes in LSEC following 24 h treatment with conditioned medium from IMR90 fibroblasts undergoing oncogene-induced senescence due to *RAS* overexpression (Ras-CM). VE-cadherin (red) indicates inter-cellular junctions and DAPI (blue) was used as a nuclear counterstain *(left).* Elongation factor (*right*) was calculated by dividing cell length by cell width (71-93 cells total). Data shown are mean ± SEM from three independent cell isolates (*$p<0.05$, student's unpaired *t*-test)

(B) Cytoskeletal rearrangement in LSEC following 24 h treatment with Ras-CM. F-actin (grey) was visualised by phalloidin staining and DAPI (blue) was used as a nuclear counterstain. Quantification of phalloidin staining (% area) shows mean ± SEM from three independent cell isolates (***$p<0.001$, student's unpaired *t*-test)

(C) Gene expression of endothelial activation markers following 24 h treatment with Ras-CM or the growing control (Grow-CM). Data shown are gene expression relative to *GAPDH* from three independent cell isolates (*$p<0.05$, student's paired *t*-test)

(D) Immunocytochemical staining for intercellular adhesion molecule 1 (ICAM-1, green) (*upper*) and CCL2 (red) (*lower*) in Ras-CM-treated HSEC compared to Grow-CM-treated controls. Perinuclear CCL2 localisation is indicated by yellow arrowheads. Quantification (% area) (*right*) shows mean ± SEM from three independent cell isolates (**$p<0.01$, student's unpaired *t*-test)

(E) Dual immunofluorescent staining of CCL2 (red) and Golgi marker, GM130 (green). DAPI (blue) was used as a nuclear counterstain. Yellow arrow depicts site of intensity profile (*lower*)

Supplementary Figure 6

A

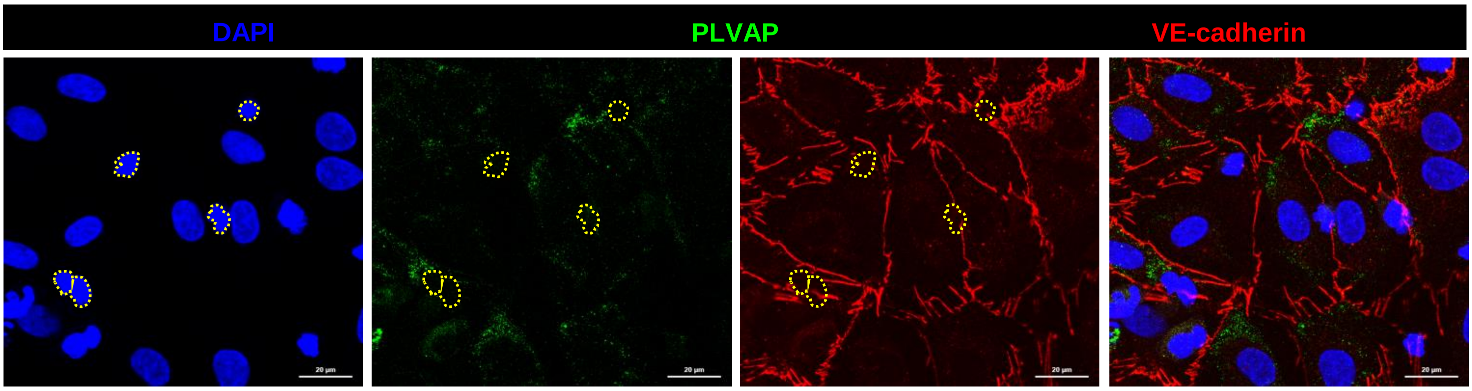

B

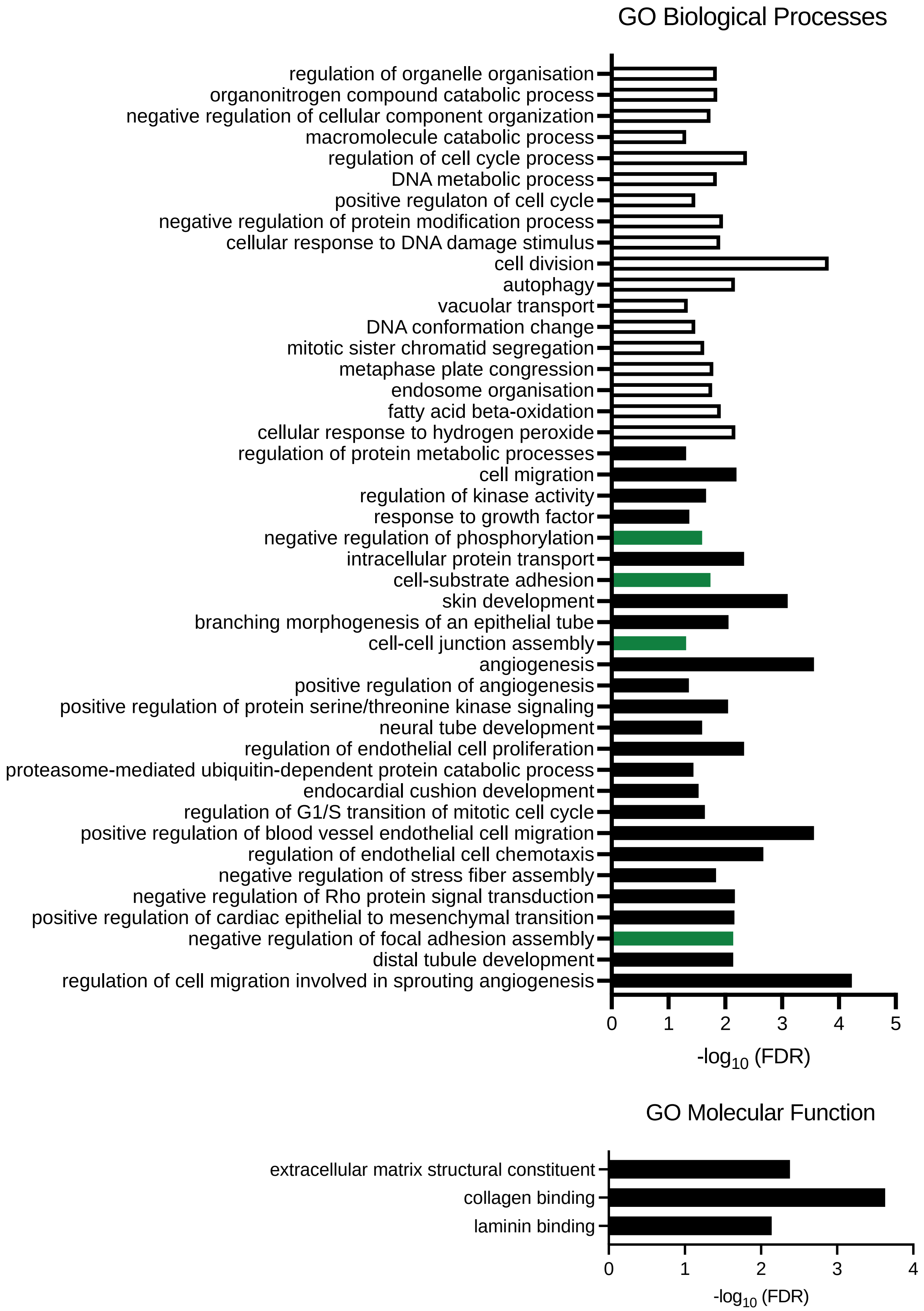

Supplementary Figure 6, Related to Figure 6

Plasmalemma vesicle-associated protein (PLVAP) may regulate paracellular permeability indirectly by altering cell-cell and cell-matrix adhesion.

(A) PLVAP (green) does not seem to specifically localise with transmigrating monocytes (yellow dashed lines) in Ras-CM treated LSEC. VE-cadherin (red) indicates intercellular junctions and DAPI (blue) was used as a nuclear counterstain

(B) Gene ontology (GO) biological processes *(upper)* and molecular function *(lower)* pathway analysis following *PLVAP* genetic knockdown in LSEC. Unfilled bars indicate downregulation and filled bars indicate upregulation in siPLVAP cells. Relevant pathways are highlighted in green

Aetiology	Age	Sex
**ALD**	66	M
**ALD**	69	M
**ALD/HCC**	68	F
**BC**	72	M
**NASH**	62	F
**PBC**	71	F
**PSC/AMR/DILI**	48	F
**SBC**	50	M

Supplementary Table 1 – LSEC isolation patient details. Patient details were anonymised according to regulations approved by South Birmingham Research Ethics Committee. ALD = Alcoholic liver disease, HCC = hepatocellular carcinoma; BC = biliary cirrhosis; NASH = Non-alcoholic steatohepatitis; PBC = Primary biliary cholangitis; PSC = Primary sclerosing cholangitis; AMR = Antibody-mediated rejection; DILI = Drug-induced liver injury; SBC = Secondary biliary cirrhosis.

	Normal liver	ALD	NASH	PBC	PSC
**Cohort (n)**	20	9	15	13	11
**% female**	-	33.33%	46.67%	76.92%	36.36%
**Age (median ± SD)**	-	68 ± 10.31	67 ± 3.14	62 ± 12.42	37 ± 16.54

Supplementary Table 2 – Patient details for liver tissue samples used throughout the study. Patient details were anonymised according to regulations approved by South Birmingham Research Ethics Committee. Normal liver/Donor details were not available, ALD = Alcoholic liver disease, NASH = Non-alcoholic steatohepatitis; PBC = Primary biliary cholangitis; PSC = Primary sclerosing cholangitis details were available due to pseudo-anonymisation and linked records.
